# Biocontrol Potential of *Aspergillus* Species Producing Antimicrobial Metabolites

**DOI:** 10.3389/fmicb.2021.804333

**Published:** 2021-12-23

**Authors:** Men Thi Ngo, Minh Van Nguyen, Jae Woo Han, Bomin Kim, Yun Kyung Kim, Myung Soo Park, Hun Kim, Gyung Ja Choi

**Affiliations:** ^1^Center for Eco-friendly New Materials, Korea Research Institute of Chemical Technology, Daejeon, South Korea; ^2^Department of Medicinal Chemistry and Pharmacology, University of Science and Technology, Daejeon, South Korea; ^3^School of Biological Sciences, Seoul National University, Seoul, South Korea

**Keywords:** *Aspergillus candidus*, *Aspergillus montenegroi*, plant disease, biocontrol, antimicrobial compound

## Abstract

Microbial metabolites have been recognized as an important source for the discovery of new antifungal agents because of their diverse chemical structures with novel modes of action. In the course of our screening for new antifungal agents from microbes, we found that culture filtrates of two fungal species *Aspergillus candidus* SFC20200425-M11 and *Aspergillus montenegroi* SFC20200425-M27 have the potentials to reduce the development of fungal plant diseases such as tomato late blight and wheat leaf rust. From these two *Aspergillus* spp., we isolated a total of seven active compounds, including two new compounds (**4** and **6**), and identified their chemical structures based on the NMR spectral analyses: sphaeropsidin A (**1**), *(R)*-formosusin A (**2**), *(R)*-variotin (**3**), candidusin (**4**), asperlin (**5**), montenegrol (**6**), and protulactone A (**7**). Based on the results of the *in vitro* bioassays of 11 plant pathogenic fungi and bacteria, sphaeropsidin A (**1**), (*R*)-formosusin A (**2**), (*R*)-variotin (**3**), and asperlin (**5**) exhibited a wide range of antimicrobial activity. Furthermore, when plants were treated with sphaeropsidin A (**1**) and (*R*)-formosusin A (**2**) at a concentration of 500 μg/ml, sphaeropsidin A (**1**) exhibited an efficacy disease control value of 96 and 90% compared to non-treated control against tomato late blight and wheat leaf rust, and (*R*)-formosusin A (**2**) strongly reduced the development of tomato gray mold by 82%. Asperlin (**5**) at a concentration of 500 μg/ml effectively controlled the development of tomato late blight and wheat leaf rust with a disease control value of 95%. Given that culture filtrates and active compounds derived from two *Aspergillus* spp. exhibited disease control efficacies, our results suggest that the *Aspergillus*-produced antifungal compounds could be useful for the development of new natural fungicides.

## Introduction

The world population is expected to reach up to 9.7 billion people by 2050, postulating that the required agricultural food production increases up to at least 50–110% (Godfray et al., 2010; Raymaekers et al., 2020). Considering that the additional agricultural area is limited, the reduction of crop yield losses caused by plant pathogens has gained the most attention contributing to food security (Mauser et al., 2015; Raymaekers et al., 2020). Although the use of synthetic pesticides has been recognized as one of the most effective methods to control plant diseases, the overuse of chemical pesticides has led to severe problems, such as resistance, toxicity in humans and animals, and environmental pollution (Hüter, 2011). To compensate for the shortcomings of chemical pesticides, the development and use of biological pesticides based on natural resources exhibiting a promising antimicrobial activity have been crucial in agriculture (Dayan et al., 2009; Choi et al., 2010).

To date, there are over 23,000 known microbial secondary metabolites that are significant sources for life-saving drugs, and 42% of which are produced by fungi (Demain, 2014; Chandra et al., 2020). The genus *Aspergillus* has been recognized as an enormous source of lead compounds with promising diverse structures and biological activities (Sadorn et al., 2016). Notably, from 2015 to 2019, 362 secondary metabolites were isolated from different *Aspergillus* species, including alkaloids, butenolides, and cytochalasins, which showed diverse biological activities such as antimicrobial, anti-inflammatory, and anticancer activities (El-hawary et al., 2020). Regarding the antimicrobial activity, it has been reported that aspetritones and candidusin derivatives isolated from the culture of *Aspergillus tritici* SP2-8-1 exhibited antibacterial activity against the methicillin-resistant strain *Staphylococcus aureus* (Wang et al., 2017). Two new compounds, aspergillethers A and B, from the endophytic fungus *Aspergillus versicolor* exhibited significant *in vitro* antibacterial and antifungal activities toward *S. aureus*, *Bacillus cereus*, and *Candida albicans* (Mohamed et al., 2020). Despite efforts to find secondary metabolites showing antimicrobial activity, to date, relatively few *Aspergillus* species have been considered as biological agents for plant protection (Zhao et al., 2018; El-Sayed and Ali, 2020).

With the production of antimicrobial metabolites, some fungal species have been reported as a potent material for controlling plant diseases. For example, the basidiomycete fungus *Crinipellis rhizomaticola* culture filtrate and its active compounds crinipellins suppressed the development of rice blast and pepper anthracnose caused by *Magnaporthe oryzae* and *Collectotrichum coccodes*, respectively (Han et al., 2018). Jang et al. (2016) also showed that a culture filtrate of *Aspergillus niger* F22 was highly active against a root-knot nematode *Meloidogyne incognita* by which a nematicidal component oxalic acid affected the mortality of second-stage juveniles and the inhibition of egg hatching. Later, the strain *A. niger* F22 was registered as a natural nematicidal agent in the Korean market (Lee et al., 2017).

The marine environment has been investigated for new natural resources containing bioactive compounds with benefits for the health of humans, animals, and plants (Sohn and Oh, 2010). For this endeavor, marine-derived resources have gained much attention in the past decades (Buttachon et al., 2018). In particular, marine-derived fungi have been considered as a rich source of secondary metabolites with promising antimicrobial effects (Neuhaus et al., 2019; Wang et al., 2021), representing unprecedented scaffolds for further drug design for specific modes of action (Xu et al., 2015; Willems et al., 2020). In the current study, our main goals were (1) to find *Aspergillus* species derived from a marine environment that have *in vitro* and *in vivo* antimicrobial properties against plant pathogens and (2) to identify the active metabolites from the selected *Aspergillus* species. Based on the *in vitro* antimicrobial activity and plant disease control efficacies of the fungal cultures containing the identified active compounds, our results could provide valuable information to develop new biological control agents for crops.

## Materials and Methods

### Fungal and Bacterial Strains Used in This Study

The eight strains of *Aspergillus* species used in this study were kindly provided by Dr. Myung Soo Park (Marine Fungal Resource Bank, Seoul National University; Supplementary Table S1). Of these strains, SFC20200425-M11 and SFC20200425-M27 showing a promising plant disease control efficacy were deposited as a patent microorganism to the Korean Agricultural Culture Collection (KACC, Wanju, South Korea).

For the *in vitro* antifungal activity assay, six plant pathogenic fungi provided by the KACC were used: *Alternaria brassicicola* (KACC 40036), *Botrytis cinerea* (KACC 48736), *C. coccodes* (KACC 48737), *Fusarium oxysporum* (KACC 40043), *M. oryzae* (KACC 46552), and *Phytophthora infestans* (KACC 48738). Additionally, we used two obligate parasitic fungi *Puccinia triticina* and *Blumeria graminis* f. sp. *hordei*, which were maintained on their host plants, for the disease control efficacy assay (Choi et al., 2010; Shin et al., 2017; Han et al., 2018). For the antibacterial activity assay, the five following bacterial species were used: *Agrobacterium tumefaciens* SL2434, *Clavibacter michiganensis* SL4135, *Pseudomonas syringae* SL308, *Ralstonia solanacearum* SL1944, and *Erwinia amylovora* TS3128. All bacteria were provided by the National Academy of Agricultural Sciences (Wanju, Korea), except for *R. solanacearum* provided by Dr. SW Lee of Dong-A University (Vu et al., 2017). The fungi and bacteria were maintained on potato dextrose agar (PDA; BD Difco, Sparks, MD, United States) medium and tryptic soy agar (TSA; BD Difco) medium, respectively, and kept at 4°C before use.

### Phylogenetic Analysis

For the isolation of genomic DNA (gDNA), each fungal species was grown in 50 ml of potato dextrose broth (PDB; BD Difco) medium at 25°C for 4 days on a rotary shaker (150 rpm). The gDNA was extracted using the cetyltrimethylammonium bromide (CTAB) procedure as previously described (Han et al., 2018). For phylogenetic analysis, the calmodulin (*CaM*) gene was amplified by the primer set CF1D (5'–CAGGTCTCCGAGTACAAG–3') and CF4 (5'–CAGGTCTCCGAGTACAAGTTTYTGCATCATRAGYTGGAC–3'; Lee et al., 2016). The resulting amplicon was purified using the GeneAll ExpinTM PCR purification kit (GeneAll, Seoul, South Korea) and then analyzed using corresponding PCR primers by Macrogen (Seoul, Korea). The resulting sequences were analyzed with the BLASTn program of the NCBI.^1^ The sequences were aligned using ClustalW implemented in MEGA version X, and distances were estimated based on the Tamura-Nei model (Tamura and Nei, 1993). A phylogenetic tree was generated using the neighbor-joining method with 1,000 bootstrap analyses (Saitou and Nei, 1987).

### Isolation Procedures of Antimicrobial Metabolites

Twenty mycelial disks (8 mm in diameter) of each fungal strain, SFC20200425-M11 and SFC20200425-M27, were inoculated into 400 ml PDB medium in a 2 L-baffled Erlenmeyer flask and incubated on a rotary shaker at 150 rpm and 25°C for 10 days. The culture broths of SFC20200425-M11 (3.6 L) and SFC20200425-M27 (1.6 L) were centrifuged at 10,000 × *g* for 30 min and then filtered through two layers of Whatman No. 1 filter paper (Maidstone, United Kingdom). The culture filtrates were partitioned with an equal volume of ethyl acetate (EtOAc) and *n*-butanol (BuOH), sequentially. Each layer was concentrated to dryness by a rotary evaporator (Rotavapor R-300; Büchi, Flawil, Switzerland).

From the culture filtrate of SFC20200425-M11, the EtOAc (370 mg), BuOH (640 mg), and water (6.8 g) extracts were obtained. The EtOAc extract was applied onto a silica gel column (40–63 μm; Merck, Darmstadt, Germany), using an isocratic elution of *n*-hexane/EtOAc (3:1, v/v) to give five fractions (E1–E5). The E3 and E4 fractions were pure compounds **1** (51 mg) and **2** (35 mg), respectively. Fraction E5 (152 mg) was further separated by preparative thin-layer chromatography (TLC) using Kieselgel 60 F254 glass plates (Merck). The TLC plates were developed with *n*-hexane/EtOAc (60:40, v/v) to give five fractions (E51–E55). Fraction E55 was a pure compound **4** (13 mg), and fraction E52 was further purified by high-pressure liquid chromatography (HPLC) using the Shimadzu LC-6 AD system (Kyoto, Japan). The Capcell Pak C18 column (20 × 250 mm, 5 μm; Shiseido, Tokyo, Japan) was used for preparative HPLC and eluted with 68% aqueous methanol at a flow rate of 5 ml/min to give compound **3** (2 mg).

Among the obtained EtOAc (338 mg), BuOH (340 mg), and water (3.1 g) extracts from the culture broth of SFC20200425-M27, the EtOAc extract was purified by preparative HPLC. The column was eluted with 30% aqueous methanol to give compound **5** (91.2 mg). Next, the BuOH extract was separated by an Isolera One mid-pressure liquid chromatography (MPLC) system (Biotage, Uppsala, Sweden) equipped with the Biotage SNAP Ultra C18 cartridge (60 g). The column was eluted with a linear gradient of aqueous methanol (2–100%, v/v) to give two fractions (B1 and B2). Fraction B2 was pure compound **7** (21 mg). The fraction B1 (70 mg) was further purified by preparative HPLC. The column was eluted with 8% aqueous methanol to give compound **6** (22 mg). The isolation schemes for all compounds **1**–**7** are presented in Supplementary Figures S1 and S2.

### General Experimental Procedures for Chemical Structural Elucidation

Chemical structures of the purified compounds were determined by spectroscopic analyses and comparison with previous literature data. High-resolution electrospray ionization mass spectrometry (HRESIMS) data were obtained by the Synapt G2 system (Waters, Milford, MA, United States). The 1D and 2D nuclear magnetic resonance (NMR) spectra were recorded by a Bruker Advance 500 MHz spectrometer (Rheinstetten, Germany) in chloroform-*d*, methanol-*d*_4_, or pyridine-*d*_5_ (Cambridge Isotope Laboratories, Andover, MA, United States). Chemical shifts were referenced to the solvent peaks (*δ*_H_ 7.26 and *δ*_C_ 77.2 for chloroform-*d*; *δ*_H_ 4.87 and *δ*_C_ 49.0 for methanol-*d*_4_; *δ*_H_ 8.71 and *δ*_C_ 149.9 for pyridine-*d*_5_).

### Esterification of Compound 4

To determine the absolute configuration of the secondary alcohol compound **4**, Mosher’s method using *α*-methoxy-*α*-trifluoromethylphenylacetic acid (MTPA) esters was performed as previously described by Nguyen et al. (2018). Briefly, the (*R*/*S*)-MTPA esters of **4** (**4a** and **4b**) were prepared using Mosher’s esterification method. Compounds **4** (0.5 mg) and 4-(dimethylamino)-pyridine (0.2 mg) were mixed into a 5 ml vial, and then, the mixture was dried *in vacuo*. Pyridine-*d_5_* (0.5 ml) and (*R*)-MPTA or (*S*)-MPTA (Sigma-Aldrich, St Louis, MO, United States; 6.0 μl) were immediately put into the vial, and then the vial was sealed and shaken to mix evenly. The reaction was carried out at room temperature for 12 h. The reactant was transferred into an NMR tube to measure the ^1^H-NMR and ^1^H–^1^H COSY spectra.

### *In vitro* Antimicrobial Assay

The values for the minimum inhibitory concentration (MIC) of the purified compounds **1**–**7** were determined against plant pathogenic fungi and bacteria by the broth microdilution method using 96-well microtiter plates modified according to previous methods for the testing of potential antimicrobial natural products (Espinel-Ingroff et al., 2005; Vu et al., 2017; Ngo et al., 2019). Briefly, fungal spore suspensions (5 × 10^4^ spores/ml) or bacterial cell suspensions (2 × 10^5^ CFU/ml) were added to each well of a 96-well microtiter plate containing PDB or tryptic soy broth (TSB; BD Difco) medium, respectively. The purified compounds dissolved in methanol were added and then serially two-fold diluted to reach the final concentrations ranging from 0.06 to 250 μg/ml. The microliter plates were incubated for 1–2 days, and the MIC values were determined by visual inspection of complete growth inhibition (Espinel-Ingroff et al., 2005). Blasticidin-S and oxytetracycline were used as positive controls for the antifungal and antibacterial assays, respectively. The medium containing 1% methanol was used as a negative control.

### Disease Control Efficacy Assay

Six plant diseases caused by fungi were used for the plant disease control efficacy assay: rice blast (RCB, caused by *M. oryzae*), tomato gray mold (TGM, caused by *B. cinerea*), tomato late blight (TLB, caused by *P. infestans*), wheat leaf rust (WLR, caused by *P. triticina*), barley powdery mildew (BPM, caused by *B. graminis* f. sp. *hordei*), and pepper anthracnose (PAN, caused by *C. coccodes*). We performed the plant disease control efficacy assay as previously described (Lee et al., 2007; Ngo et al., 2021). Briefly, rice (*Oryza sativa* L, cv. Chucheong), tomato (*Solanum lycopersicum* cv. Seokwang), wheat (*Triticum aestivum* cv. Geumgang), barley (*Hordeum sativum* cv. Hanyoung), and pepper (*Capsicum annuum* cv. Hyangchon) were used as host plants, which were grown in a greenhouse at 25 ± 5°C for 3–4 weeks. One day before pathogen inoculation, the culture filtrates were directly applied onto the plant by spraying. The plants were also treated with the solvent extracts (1,000 μg/ml) and pure compounds (125, 250, and 500 μg/ml), which were dissolved in 5% aqueous methanol (v/v), using the same method for the culture filtrates. When the culture filtrates, solvent extracts, and pure compounds were applied to the plants, the samples contained 0.025% Tween 20 (w/v) as a wetting agent. Chemical fungicides (blasticidin S, validamycin, fludioxonil, dimethomorph, flusilazole, and dithianon) and 5% aqueous methanol were used as positive and negative controls, respectively. The treated plants were inoculated with spore suspensions (5 × 10^4^ spores/ml) of each fungal pathogen and incubated as previously described (Ngo et al., 2021). The experiment was conducted twice with three replicates for each treatment. The disease control efficacy was calculated with the following equation: control efficacy (%) = 100 × [1 − B/A], where A is the mean lesion area (%) on the leaves of the control plants, and B is the mean lesion area (%) on the leaves of the treated plants (Lee et al., 2007).

### Statistical Analysis

Data were subjected to one-way ANOVA, and the means of the treatments were separated by Duncan’s multiple range test (*p* < 0.05) using the *R*-software (Version 4.0.5). All values are expressed as the mean ± standard deviation. Significant differences (*p* < 0.05) were indicated with different small letters in each bar.

## Results and Discussion

### Identification of the Marine-Derived *Aspergillus* Species

**Figure 1:**
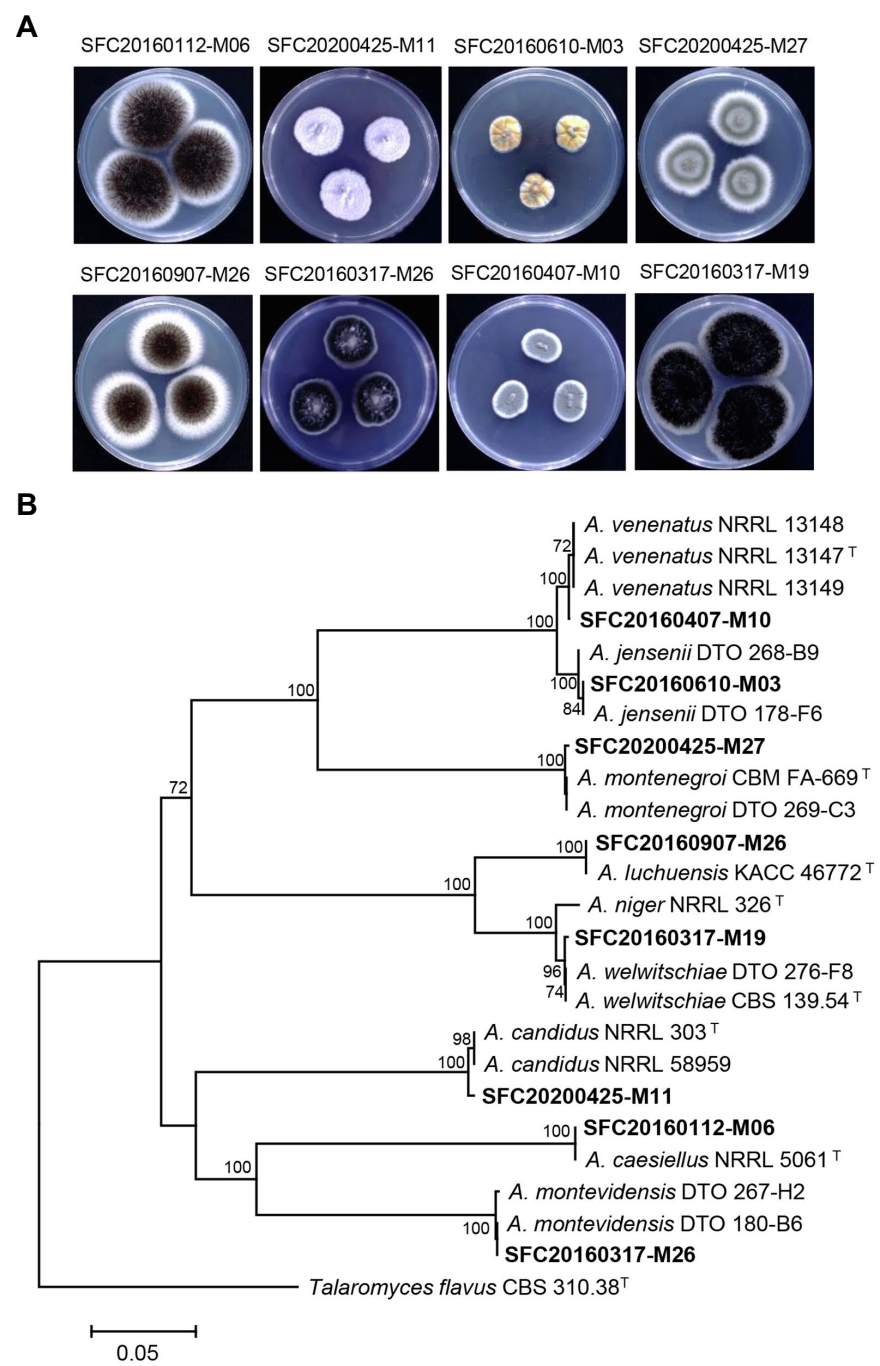
The colony morphology **(A)** and phylogenetic tree **(B)** of eight *Aspergillus* strains isolated from different sites of the Korean coast. *Aspergillus* isolates were cultivated on potato dextrose agar medium at 25°C for 7 days. The calmodulin gene (*CaM*)-based phylogenetic analysis was performed using a neighbor-joining method with 1,000 bootstrap samplings. Bootstrap scores (>70%) are presented at the nodes. The scale bar indicates the number of nucleotide substitutions per site. T indicates type strains.

Marine-derived fungi have been considered as a rich source of bioactive compounds with promising antimicrobial effects and plant disease control efficacy (Xu et al., 2015; Wang et al., 2021). In this study, a total of eight marine-derived *Aspergillus* spp. was isolated from different sites of the Korean coast (Supplementary Table S1). After cultivation on PDA medium for 7 days, eight strains of marine-derived *Aspergillus* showed different colony morphologies (Figure 1A). The *CaM* gene-based phylogenetic tree was constructed for the identification of the *Aspergillus* species (Figure 1B). In a previous study, two *Aspergillus* strains SFC20160112-M06 and SFC20160407-M10 were identified as *Aspergillus caesiellus* and *A. venenatus*, respectively (Lee et al., 2016). Here, six *Aspergillus* strains SFC20160610-M03, SFC20200425-M27, SFC20160907-M26, SFC20160317-M19, SFC20200425-M11, and SFC20160317-M26 were identified as *A. jensenii*, *A. montenegroi*, *A. luchuensis*, *A. welwitschiae*, *A. candidus*, and *A. montevidensis*, respectively (Figure 1B).

### Plant Disease Control Efficacy of the Culture Filtrates and Their Partitioned Fractions

To explore the potential of these *Aspergillus* strains in plant disease control, the disease control efficacy of the culture filtrates was investigated against six plant diseases RCB, TGM, TLB, WLR, BPM, and PAN (Table 1). When the plants were treated with each culture filtrate, the culture filtrate of *A. candidus* SFC20200425-M11 significantly reduced the disease development of TGM and PAN with control values of 82 and 91%, respectively, compared to the non-treatment control (Table 1). In contrast, there were weak or no effects on RCB, TLB, WLR, and BPM. The culture filtrate of *A. montenegroi* SFC20200425-M27 exhibited disease control values of 98 and 100% for TLB and WLR, respectively, whereas the development of RCB, TGM, BPM, and PAN were weak or not inhibited by this culture filtrate (Table 1). The culture filtrates of *A. caesiellus* SFC20160112-M06, *A. jensenii* SFC20160610-M03, and *A. montevidensis* SFC20160317-M26 showed weak or no disease control efficacies (Table 1). Moreover, the culture filtrates of *A. luchuensis* SFC20160907-M26, *A. venenatus* SFC20160407-M10, and *A. welwitschiae* SFC20160317-M19 exhibited phytotoxic symptoms on the foliage parts of the plants (Table 1). Thus, among the eight *Aspergillus* strains, *A. candidus* SFC20200425-M11 and *A. montenegroi* SFC20200425-M27 were selected as potential biocontrol agents in which they may produce antifungal compounds for plant diseases control.

To identify active compounds in the culture filtrates, we explored the disease control efficacy of the solvent extracts derived from the culture filtrates. When each extract was sprayed at a concentration of 1,000 μg/ml onto the plants 24 h before the inoculation of the fungal pathogens, the EtOAc extract of *A. candidus* SFC20200425-M11 exhibited a broad spectrum of disease control efficacy against TGM, TLB, WLR, and PAN with control values of 100, 94, 73, and 91%, respectively, compared to the non-treatment controls (Table 1). However, the BuOH and water extracts had no significant effects on all tested plant diseases (Table 1). These results suggest that the EtOAc extract of *A. candidus* SFC20200425-M11 might contain antifungal substances.

In contrast to the solvent extracts of the *A. candidus* culture, both the EtOAc and BuOH extracts of *A. montenegroi* SFC20200425-M27 effectively controlled plant diseases. The EtOAc showed control values of 79, 84, 100, and 85% against RCB, TLB, WLR, and PAN, respectively (Table 1). The BuOH extract also exhibited disease control efficacies against RCB, TLB, WLR, and PAN with control values of 85, 82, 100, and 85%, respectively (Table 1). The water extract had no effect on all tested plant diseases (Table 1). These results suggest that *A. candidus* SFC20200425-M11 and *A. montenegroi* SFC20200425-M27 produce antifungal substances that have lipophilic properties.

**Table 1:** *In vivo* antifungal activity of *Aspergillus* species against plant pathogenic fungi.

Treatment	Conc. (μg/ml)	Disease control (%)					
		RCB	TGM	TLB	WLR	BPM	PAN
*Aspergillus caesiellus* (SFC20160112-M06)	*cf*	0^e^	0^d^	0^c^	0^d^	0^e^	10 ± 7^c^
*A. candidus* (SFC20200425-M11)	*cf*	0^e^	82 ± 5^b^	36 ± 10^b^	20 ± 0^c^	25 ± 12^cd^	91 ± 5^a^
*A. jensenii* (SFC20160610-M03)	*cf*	17 ± 13^de^	0^d^	0^c^	60 ± 9^b^	33 ± 0^c^	0^c^
*A. luchuensis* (SFC20160907-M26)	*cf*	pt	0^d^	0^c^	pt	pt	75 ± 9^a^
*A. montenegroi* (SFC20200425-M27)	*cf*	33 ± 0^d^	0^d^	98 ± 1^a^	100^a^	0^e^	35 ± 7^b^
*A. montevidensis* (SFC20160317-M26)	*cf*	0^e^	0^d^	0^c^	0^d^	0^e^	10 ± 7^c^
*A. venenatus* (SFC20160407-M10)	*cf*	pt	pt	pt	pt	pt	pt
*A. welwitschiae* (SFC20160317-M19)	*cf*	pt	pt	pt	pt	pt	pt
*A. candidus* EtOAc extract	1,000	56 ± 9^c^	100^a^	94 ± 2^a^	73 ± 9^b^	8 ± 6^de^	91 ± 5^a^
*A. candidus* BuOH extract	1,000	0^e^	21 ± 10^c^	0^c^	20 ± 0^c^	0^e^	0^c^
*A. candidus* water extract	1,000	0^e^	0^d^	0^c^	0^d^	0^e^	0^c^
*A. montenegroi* EtOAc extract	1,000	79 ± 5^abc^	7 ± 5^cd^	84 ± 10^a^	100^a^	58 ± 12^b^	85 ± 4^a^
*A. montenegroi* BuOH extract	1,000	85 ± 4^ab^	0^d^	82 ± 5^a^	100^a^	8 ± 6^de^	85 ± 4^a^
*A. montenegroi* water extract	1,000	0^e^	0^d^	0^c^	0^d^	0^e^	0^c^
Blasticidin-S	1	69 ± 9^bc^	–	–	–	–	–
	50	100^a^	–	–	–	–	–
Fenhexamide	20	–	94 ± 2^ab^	–	–	–	–
	100	–	100^a^	–	–	–	–
Dimethomorph	2	–	–	50 ± 10^b^	–	–	–
	10	–	–	100^a^	–	–	–
Flusilazole	2	–	–	–	60 ± 9^b^	–	–
	10	–	–	–	100^a^	–	–
Benomyl	20	–	–	–	–	77 ± 0^b^	–
	100	–	–	–	–	100^a^	–
Dithianon	10	–	–	–	–	–	11 ± 7^c^
	50	–	–	–	–	–	93 ± 1^a^

*Disease control values (%) represent the mean of three replicates. Values with different letters are significantly different at p < 0.05 according to Duncan’s multiple range test. RCB, rice blast; TGM, tomato gray mold; TLB, tomato late blight; WLR, wheat leaf rust; BPM, barley powdery mildew; PAN, pepper anthracnose; cf, culture filtrate; pt, phytotoxicity; and –, not tested. The values represent the mean ± standard deviation of two runs with three replicates. Different small letters in each column indicate a significant difference at p < 0.05*.

### Chemical Identification of the Active Metabolites

**Figure 2:**
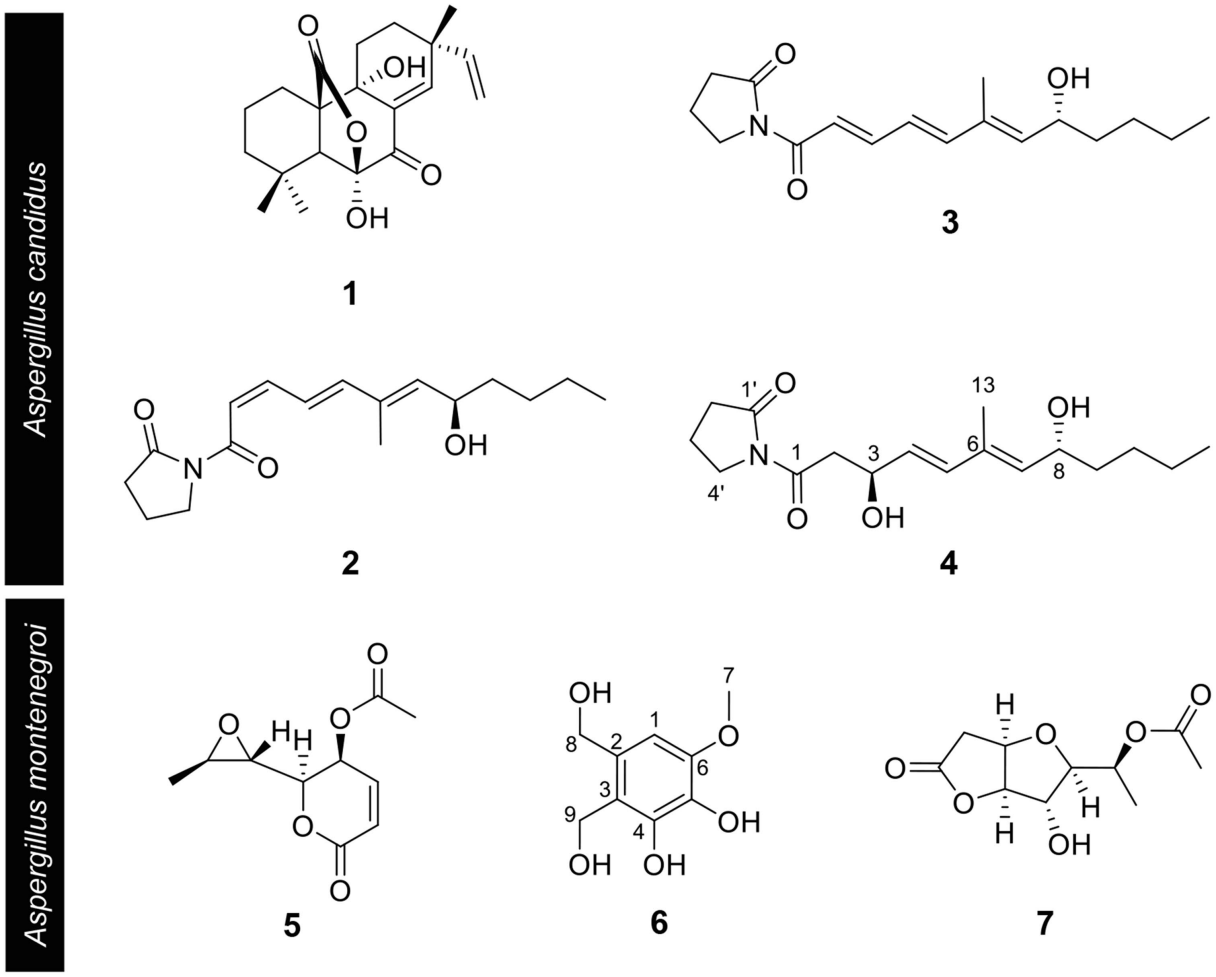
Chemical structures of compounds **1**–**7** isolated from *Aspergillus candidus* SFC20200425-M11 and *Aspergillus montenegroi* SFC20200425-M27. Sphaeropsidin A (**1**), *(R)*-formosusin A (**2**), (*R*)-variotin (**3**), and candidusin (**4**) were isolated from *Aspergillus candidus*. Asperlin (**5**), montenegrol (**6**), and protulactone A (**7**) were isolated from *A. montenegroi*.

Based on the results of the disease control efficacy assays, the EtOAc extract of *A. candidus* SFC20200425-M11 and the EtOAc and BuOH extracts of *A. montenegroi* SFC20200425-M27 were further separated by various chromatographic procedures with the guidance of *in vitro* antifungal assays against *B. cinerea* or *P. infestans*. Compounds **1**–**4** were isolated from the EtOAc extract of *A. candidus* SFC20200425-M11, and compounds **5**–**7** were isolated from the EtOAc and BuOH extracts of *A. montenegroi* SFC20200425-M27. By comparing our spectroscopic data (Supplementary Table S2) of the isolated compounds with those reported in the literature (Yonehara et al., 1959; Ellestad et al., 1972; Mizuba et al., 1975; Sohn and Oh, 2010; Mizushina et al., 2014), five of them were identified as known compounds: sphaeropsidin A (**1**), *(R)*-formosusin A (**2**), *(R)*-variotin (**3**), asperlin (**5**), and protulactone A (**7**). For the structural determination of the new compounds **4** and **6**, MS and NMR spectroscopic analyses were performed (Table 2; Supplementary Figures S3–S17). All the chemical structures of compounds **1**–**7** are shown in Figure 2.

**Table 2:** The ^1^H and ^13^C NMR data (500 and 125 MHz) for new compounds **4** and **6**.

Position	Candidusin (**4**) in chloroform-*d*		Motenegrol (**6**) in methanol-*d*_4_	
	*δ*_H_, (*J* in Hz)	*δ*_C_, Type	*δ*_H_, (*J* in Hz)	*δ*_C_, Type
1	–	173.1, CO	6.53, s	104.9, CH
2	3.13, d (4.0); 3.12, d (8.3)	44.1, CH_2_	–	132.2, C
3	4.66, m	68.6, CH	–	119.9, C
4	5.69, dd (15.7, 6.3)	129.5, CH	–	145.8, C
5	6.27, d (15.7)	135.0, CH	–	134.5, C
6	–	134.3, C	–	148.6, C
7	5.42, d (8.7)	135.4, CH	3.81, s	56.4, CH_3_
8	4.43, q (6.7)	68.5, CH	4.58, s	63.6, CH_2_
9	1.58, m; 1.42, m	37.3, CH_2_	4.71, s	56.6, CH_2_
10	1.28^*^	27.5, CH_2_	–	–
11	1.28^*^	22.7, CH_2_	–	–
12	0.86, t (7.0)	14.1, CH_3_	–	–
13	1.76, d (1.2)	13.0, CH_3_	–	–
1'	–	175.8, CO	–	–
2'	2.58, t (8.1)	33.6, CH_2_	–	–
3'	2.02, m	17.2, CH_2_	–	–
4'	3.79, t (7.2)	45.4, CH_2_	–	-

All proton and carbon positions are assigned by ^1^H–^1^H COSY, HSQC, and HMBC experiments. Em dashes indicate “not detected.”

* (Asterisks) indicate overlapped signals.

**Figure 3:**
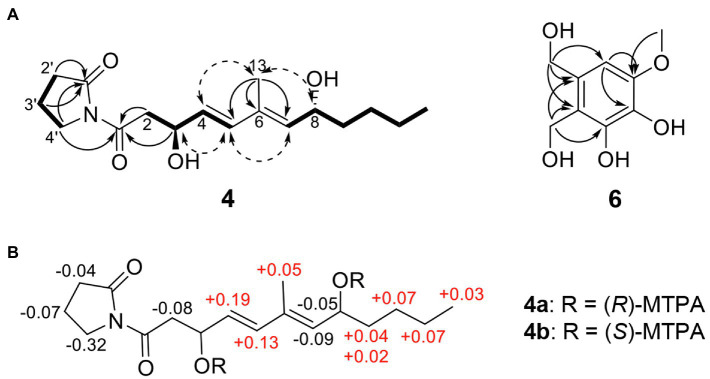
Structural identification of new compounds. **(A)** Key HMBC (arrow), COSY (bold line), and NOESY (dotted arrow) correlations of compounds **4** and **6**. **(B)** The Δ *δ_S_* – *δ_R_* values for (*R*)- and (*S*)-MTPA esters of compound **4**.

The HRESIMS spectrum of compound **4** showed a quasi-molecular ion at *m/z* 332.1833 [M + Na]^+^, which was consistent with the molecular formula C_17_H_27_NO_4_ (calculated *m/z* 332.1838 for C_17_H_27_NO_4_Na; Supplementary Figure S3). The ^13^C-NMR and HSQC spectra revealed two methyls (*δ*_C_ 13.0 and 14.1), seven *sp^3^* methylenes (*δ*_C_ 17.2, 22.7, 27.5, 33.6, 37.3, 44.1, and 45.4), three olefinic methines (*δ*_C_ 129.5, 135.0, and 135.4), two oxygenated methines (*δ*_C_ 68.5, 68.6), one olefinic quaternary carbon (*δ*_C_ 134.3), and two ketone carbons (*δ*_C_ 173.1 and 175.8; Table 2; Supplementary Figures S4–S6). The *N*-substituted *γ*-lactam moiety of compound **4** was confirmed by ^1^H–^1^H COSY correlations among H-2′, H-3′, and H-4′ along with the HMBC correlations from H-2′, H-3′, and H-4′ to C-1′ (Supplementary Figures S7 and S8). It was also confirmed by the HMBC correlations from H-13 to C-5, C-6, and C-7, together with ^1^H–^1^H COSY correlations among H-2, H-3, H-4, and H-5 and among H-7, H-8, H-9, H-10, H-11, and H-12. Two parts of the structure were connected through ketone carbon C-1, which was confirmed by the correlations in the HMBC experiment from H-2, H-3, and H-4′ to C-1. The coupling constants between H-4 and H-5 (*J* = 15.7 Hz) indicated the *trans* (*E*) geometry of the double bond (Table 2). Another double bond was determined to be *E* based on NOESY correlations from H-4 to H-13 and from H-5 to H-7 (Figure 3A). The planar structure of compound **4** was similar to that of *R-*variotin (**3**), except for the presence of one aliphatic methylene at C-2 and a hydroxyl group at C-3 instead of the double bond at C-2. The absolute configuration at C-3 and C-8 was established by the NOESY experiment and Mosher’s method (Supplementary Figures S9–S13). In the NOESY experiment, correlations were observed from H-5 to H-3, from H-5 to H-7, from H-13 to H-4, and from H-13 to H-8 (Figure 3A). The secondary alcohol groups were reacted with *R*-(−)- and *S*-(+)-*α*-methoxy-*α*-(trifluoromethyl)phenylacetyl chloride (MTPA) to give *S*- and *R*-MTPA esters, **4a** and **4b**, respectively (Figure 3B). The ^1^H NMR chemical shift differences (Δ*δ _S - R_*) between **4a** and **4b** are shown in Figure 3B. Positive Δ*δ _S - R_* values for H-4 and H-5 together with negative Δ*δ _S - R_* values for H-2′, H-3′, H-4′, and H-2 indicated the *S* configuration of C-3 (Figure 3B). The absolute configuration of C-8 was determined as *R* based on positive Δ*δ _S - R_* values for H-9, H-10, H-11, and H-12 and a negative Δ*δ _S - R_* value for H-7 (Figure 3B). Thus, the structure of **4** was elucidated as 1-((3*S*,4*E*,6*E*,8*R*)-3,8-dihydroxy-6-methyldodeca-4,6-dienoyl)pyrrolidin-2-one and designated as candidusin.

The molecular formula of compound **6** was determined to be C_9_H_12_O_5_ based on the HRESIMS peaks of *m/z* 223.0581 [M + Na]^+^ and 205.0476 [M + Na − H_2_O]^+^ (calculated 223.0582 and 205.0477 for C_9_H_12_O_5_Na and C_9_H_10_O_4_Na; Supplementary Figure S14). The ^1^H NMR spectrum of compound **6** exhibited signals for one aromatic proton at *δ*_H_ 6.53, two oxygenated methylene groups at *δ*_H_ 4.71 and 4.58, and one methoxy group at *δ*_H_ 3.81 (Table 2; Supplementary Figure S15). The ^13^C NMR spectrum of compound **6** contained nine signals, including one methoxy (*δ*_C_ 56.4), two oxygenated methylenes (*δ*_C_ 56.6 and 63.6), one aromatic methine (*δ*_C_ 104.9), and five quaternary aromatic carbons (*δ*_C_ 119.9, 132.2, 134.5, 145.8, and 148.6; Table 2; Supplementary Figure S16). These data suggest that compound **6** has a penta-substituted benzene ring. The positions of five substituents were determined by the HMBC correlations as follows: from an oxygenated methylene proton H-8 (*δ*_H_ 4.58) to C-1, C-2, and C-3; from an oxygenated methylene proton H-9 (*δ*_H_ 4.73) to C-2, C-3, and C-4; from an aromatic methine proton H-1 (*δ*_H_ 6.53) to C-5 and C-6; and a methoxy proton H-7 (*δ*_H_ 3.81) to C-6 (Supplementary Figure S17). Those data suggested that the substituted two oxygenated methylenes, two hydroxyls, and one methoxy group were placed on C-2, C-3, C-4, C-5, and C-6, respectively. Therefore, the structure of compound **6** was elucidated as 2,3-dihydroxymethyl-4,5-dihydroxy-6-methoxybenzene and named montenegrol.

Candidusin (**4**): colorless oil; ${\left[\alpha \right]}_{D}^{23}$ = −10 (*c* = 0.1, methanol); IR (ATR) *ν*_max_/cm: 3,371, 2,972, 2,926, 1730, 1,645, 1,549, 1,439, 1,369, 1,242, and 1,173; ^1^H- and ^13^C-NMR (500 and 125 MHz, chloroform-*d*) data: see Table 2; Supplementary Figures S4 and S5; HRESIMS: *m/z* 332.1833 [M + Na]^+^ (calculated m/z 332.1838 for C_17_H_27_NO_4_Na; Supplementary Figure S3).

(*S*)-MTPA ester of **4** (**4a**): ^1^H NMR (Pyridine-*d_5_*, 500 MHz) δ_H_ 6.6244 (H-5), 6.0912 (H-4), 5.9078 (H-8), 5.4669 (H-7), 3.3667 (H-2), 3.1911 (H-4′), 2.4020 (H-2′), 1.9070 (H-13), 1.9069 (H-3′), 1.7134 (H-9a), 1.5270 (H-9b), 1.1980 (H-10, H-11), and 0.7707 (H-12).

(*R*)-MTPA ester of **4** (**4b**): ^1^H NMR (Pyridine-*d_5_*, 500 MHz): δ_H_ 6.4908 (H-5), 5.9625 (H-8), 5.9035 (H-4), 5.5533 (H-7), 3.5116 (H-4′), 3.4503 (H-2), 2.4464 (H-2′), 1.9755 (H-3′), 1.8536 (H-13), 1.6724 (H-9a), 1.5073 (H-9b), 1.1267 (H-10, H-11), and 0.7374 (H-12).

Motenegrol (**6**): yellow oil; ^1^H- and ^13^C-NMR (500 and 125 MHz, methanol-*d*_4_) data: see Table 2; Supplementary Figures S15 and S16; HRESIMS: *m/z* 223.0687 [M + Na]^+^ (calculated m/z 223.0582 for C_9_H_12_O_5_Na; Supplementary Figure S14).

### *In vitro* Antimicrobial Activity of the Pure Compounds 1–7

The isolated compounds **1**–**7** were evaluated for their *in vitro* antifungal and antibacterial property against six plant pathogenic fungi (*A. brassicicola*, *B. cinerea*, *C. coccodes*, *F. oxysporum*, *M. oryzae*, and *P. infestans*) and five plant pathogenic bacteria (*A. tumefaciens*, *C. michiganensis*, *P. syringae*, *R. solanacearum*, and *E. amylovora*). All MIC values of the isolated compounds **1**–**7** are presented in Table 3. Of compounds **1**–**4** identified from the culture filtrate of *A. candidus* SFC20200425-M11, sphaeropsidin A (**1**), *(R)*-formosusin A (**2**), and *(R)*-variotin (**3**) exhibited an antifungal activity against all tested fungal pathogens, but not for the bacterial pathogens. In particular, sphaeropsidin A (**1**) exhibited promising antifungal activities against *P. infestans*, *C. coccodes*, *A. brassicicola*, and *B. cinerea* with MIC values of 0.3, 8, 16, and 63 μg/ml, respectively. *(R)*-formosusin A (**2**) showed the most potential antifungal activity against *C. coccodes* with a MIC value of 1 μg/ml, followed by *B. cinerea*, *M. oryzae*, *F. oxysporum*, and *A. brassicicola* with MIC values of 4, 4, 16, and 16 μg/ml, respectively. *(R)*-variotin (**3**) exhibited promising antifungal activities against *C. coccodes*, *A. brassicicola*, and *B. cinerea* with MIC values of 8, 16, and 16 μg/ml, respectively. Among the fungal pathogens tested, *C. coccodes* was the most sensitive to sphaeropsidin A (**1**), *(R)*-formosusin A (**2**), and *(R)*-variotin (**3**) with MIC values ranging from 1 to 8 μg/ml. Despite the structural similarity with formosusin A (**2**) and *(R)*-variotin (**3**), the new compound candidusin (**4**) exclusively showed a moderate antifungal activity against *A. brassicicola* with a MIC value of 250 μg/ml.

**Table 3:** *In vitro* antimicrobial activity of compounds **1**–**7** against plant pathogen.

Plant Pathogen		MIC (μg/mL)						
		**1**	**2**	**3**	**4**	**5**	**6**	**7**
Fungus	*Alternaria brassicicola*	16	16	16	250	–	–	–
	*Botrytis cinerea*	63	4	16	–	–	–	–
	*Colletotrichum coccodes*	8	1	8	–	250	–	–
	*Fusarium oxysporum*	250	16	63	–	–	–	–
	*Magnaporthe oryzae*	125	4	125	–	31	–	–
	*Phytophthora infestans*	0.3	250	250	–	1	250	125
Bacterium	*Agrobacterium tumefaciens*	–	–	–	–	–	–	–
	*Clavibacter michiganensis*	–	–	–	–	125	–	–
	*Pseudomonas syringae*	–	–	–	–	–	–	–
	*Ralstonia solanacearum*	–	–	–	–	–	–	–
	*Erwinia amylovora*	–	–	–	–	250	–	–

***1**, sphaeropsidin A; **2**, (R)-formosusin A; **3**, (R)-variotin; **4**, candidusin; **5**, asperlin; **6**, montenegrol; **7**, protulactone A. Em dashes indicate MIC > 250 μg/ml*.

Of three isolated compounds **5**–**7** from the culture filtrate of *A. montenegroi* SFC20200425-M27, asperlin (**5**) was the most effective in suppressing the growth of *P. infestans*, *M. oryzae*, and *C. coccodes* (MICs = 1, 31, and 250 μg/ml, respectively). Montenegrol (**6**) and protulactone A (**7**) showed a moderate activity to inhibit the growth of *P. infestants* with MIC values of 250 and 125 μg/ml, respectively. Intriguingly, among all the tested compounds in this study, only asperlin (**5**) showed an antibacterial activity against *C. michiganensis* and *E. amylovora* with MIC values of 125 and 250 μg/ml, respectively.

### Plant Disease Control Efficacy of Active Compounds

**Figure 4:**
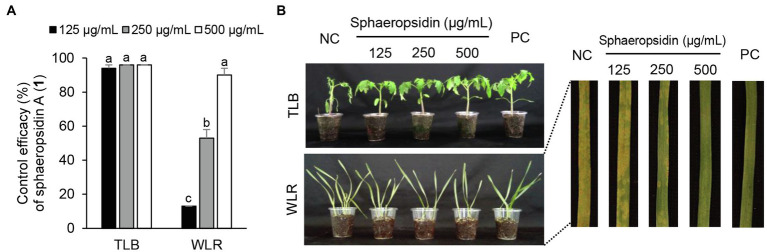
Effects of sphaeropsidin A (**1**) isolated from *Aspergillus candidus* SFC20200425-M11 on the development of tomato late blight (TLB) and wheat leaf rust (WLR) caused by *Phytophthora infestans* and *Puccinia triticina*. **(A)** Control efficacy of sphaeropsidin A (**1**) against TLB and WLR. The bars represent the mean ± standard deviation of two runs with three replicates. Different small letters in each bar indicate a significant difference at *p* < 0.05 (Duncan’s multiple range test). **(B)** Representatives of plants treated with sphaeropsidin (**1**) at a concentration of 125, 250, and 500 μg/ml. Plants were inoculated with sporangia or spores of *P. infestans* or *P. triticina* 1 day after treatment with sphaeropsidin (**1**). Treatment with Tween 20 solution containing 5% methanol and chemical fungicides (dimethomorph for TLB and flusilazole for WLR) were prepared as negative and positive controls (NC and PC), respectively.

**Figure 5:**
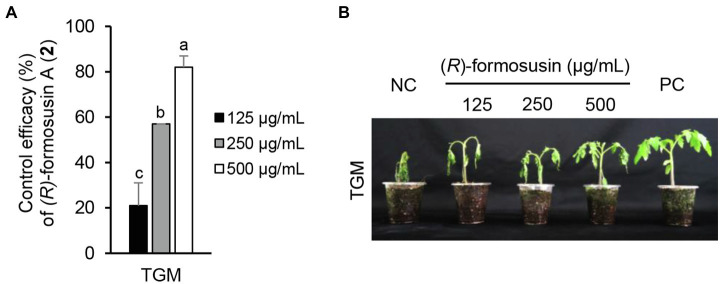
Effect of (*R*)-formosusin (**2**) isolated from *Aspergillus candidus* SFC20200425-M11 on the development of tomato gray mold (TGM) caused by *Botrytis cinerea*. **(A)** Control efficacy of (*R*)-formosusin (**2**) against TGM. The bars represent the mean ± standard deviation of two runs with three replicates. Different small letters in each bar indicate a significant difference at *p* < 0.05 (Duncan’s multiple range test). **(B)** Representatives of plants treated with (*R*)-formosusin (**2**) at a concentration of 125, 250, and 500 μg/ml. Plants were inoculated with spores of *B. cinerea* 1 day after treatment with (*R*)-formosusin (**2**). Treatment with the Tween 20 solution containing 5% methanol and a chemical fungicide (fenhexamide) were prepared as negative and positive controls (NC and PC), respectively.

**Figure 6:**
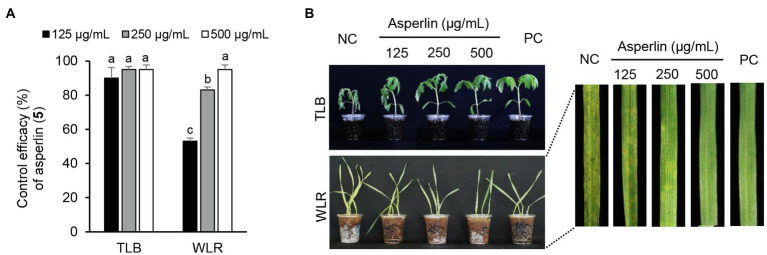
Effects of asperlin (**5**) isolated from *Aspergillus montenegroi* SFC20200425-M27 on the development of tomato late blight (TLB) and wheat leaf rust (WLR) caused by *Phytophthora infestans* and *Puccinia triticina*. **(A)** Control efficacy of asperlin (**5**) against TLB and WLR. The bars represent the mean ± standard deviation of two runs with three replicates. Different small letters in each bar indicate a significant difference at *p* < 0.05 (Duncan’s multiple range test). **(B)** Representatives of plants treated with asperlin (**5**) at a concentration of 125, 250, and 500 μg/ml. Plants were inoculated with sporangia or spores of *P. infestans* or *P. triticina* 1 day after treatment with asperlin (**5**). Treatment with Tween 20 solution containing 5% methanol and chemical fungicides (dimethomorph for TLB and flusilazole for WLR) were prepared as negative and positive controls (NC and PC), respectively.

Considering the *in vitro* antimicrobial activity and yield of the isolated compounds, we examined the disease control efficacy of sphaeropsidin A (**1**), *(R)*-formosusin A (**2**), and asperlin (**5**). When plants were treated with each compound, sphaeropsidin A (**1**) strongly reduced the disease development of TLB by at least 94% at all the treated concentrations compared to the non-treatment control. Sphaeropsidin A (**1**) also exhibited disease control values of 53 and 90% against WLR at a concentration of 250 and 500 μg/ml, respectively (Figure 4). Despite these vigorous disease control activities against TLB and WLR, sphaeropsidin A (**1**) showed weak or no disease control effects against RCB, TGM, BPM, and PAN at a high concentration of 500 μg/ml (Supplementary Table S3). In the case of *(R)*-formosusin A (**2**), it reduced in a concentration dependent manner the development of TGM with control values of 21, 57, and 82% at concentrations of 125, 250, and 500 μg/ml, respectively (Figure 5). Similar to sphaeropsidin A (**1**), asperlin (**5**) also reduced the development of TLB with control values of more than 90% at concentrations of 125–500 μg/ml and exhibited disease control values of 83 and 95% against WLR at the concentration of 250 and 500 μg/ml, respectively (Figure 6). In addition to the plant disease control efficacies, there were no phytotoxic symptoms observed on the treated plants (data not shown).

### Major Active Compounds of *Aspergillus candidus* SFC20200425-M11 and *Aspergillus montenegroi* SFC20200425-M27

In the current study, sphaeropsidin A (**1**) was isolated as one of the major antifungal compounds produced by *A. candidus* SFC20200425-M11. Sphaeropsidin A (**1**), an unrearranged pimarane diterpene, was first isolated from the fermentation of the fungus *Aspergillus chevalieri* (Ellestad et al., 1972). Since then, sphaeropsidin A (**1**) and its derivatives have been identified from various fungal species such as *Aspergilllus porosus*, *Sphaeropsis sapinea* f. sp. *cupressi*, and *Diplodia* spp. (Evidente et al., 1997; Neuhaus et al., 2019). In terms of antimicrobial activity, there were several studies presenting the potent antifungal activity of sphaeropsidin A (**1**) against plant and human pathogenic fungi (Evidente et al., 1996, 1997; Sparapano et al., 2004; Weber et al., 2007). Based on the structure–activity relationship studies, Sparapano et al. (2004) showed that the tricyclic pimarane system (particularly C-ring) and the vinyl group at C-13 are essential for the activity of sphaeropsidin A. In this study, we also showed that sphaeropsidin A (**1**) exhibits *in vitro* and *in vivo* antifungal activity against plant pathogenic fungi (Table 3; Figure 4). To the best of our knowledge, this is the first study to present the *in vivo* disease control efficacy of sphaeropsidin A (**1**) against TLB by *P. infestans* and WLR by *P. triticina* (Figure 4). Beyond the antifungal activity of sphaeropsidin A (**1**), it has also been reported to be effective against plant and human pathogenic bacteria such as *Staphylococcus haemolyticus*, *Pseudomonas aeruginosa*, and *Xanthomonas oryzae* pv. *oryzae* (Evidente et al., 2011; Roscetto et al., 2020). However, in this study, no antibacterial activity of sphaeropsidin A (**1**) was observed against plant pathogenic bacteria at a concentration of 250 μg/ml.

The other active compounds of *A. candidus* SFC20200425-M11 were (*R*)-formosusin A (**2**) and (*R*)-variotin (**3**). (*R*)-formosusin A (**2**) is a *cis*-olefin analog of (*R*)-variotin (**3**; Yonehara et al., 1959; Omolo et al., 2000; Mizushina et al., 2014; Yajima et al., 2016). (*R*)-formosusin A (**2**) was first isolated from *Paecilomyces formosus* (Mizushina et al., 2014), and its absolute configuration of (*R*)-formosusin A (**2**) was established by Yajima et al. (2016). (*R*)-formosusin A (**2**) has been known to inhibit a mammalian DNA polymerase involved in the DNA repair pathway (Beard and Wilson, 2006; Mizushina et al., 2014), but its antifungal activity has not been investigated. In contrast, (*R*)-variotin (**3**) has been reported as a broad-spectrum antifungal compound (Yonehara et al., 1959). Considering the structural similarity of (*R*)-formosusin A (**2**) and (*R*)-variotin (**3**), it may be evident that (*R*)-formosusin A (**2**) exhibits an antifungal activity against plant pathogenic fungi. As expected, we found that (*R*)-formosusin A (**2**) has an antifungal activity against plant pathogenic fungi, and it was more active than (*R*)-variotin (**3**) against *B. cinerea*, *C. coccodes*, and *M. oryzae* (Table 3). However, although the new derivative candidusin (**4**) in this study was structurally similar to (*R*)-formosusin A (**2**) and (*R*)-variotin (**3**) containing an enone moiety, we did not observe an antifungal activity at a concentration of 250 μg/ml, except for *A. brassicicola* (Table 3). This observation could be supported by the antimicrobial results of the diarylheptanoids from the black and green alder, which showed more significant activity of compounds containing the enone moiety in the aliphatic chain (Novaković et al., 2015).

Of the isolated compounds **5**–**7** from the culture broth of *A. montenegroi* SFC20200425-M27, asperlin (**5**) showed the most potent activity against the plant pathogenic fungi *P. infestans* and *M. oryzae*, and against the plant pathogenic bacteria *C. michiganensis* and *E. amylovora* (Table 3). Asperlin (**5**) is a polyketide antibiotic that was first isolated from *Aspergillu nidulans*, and its various biological activities such as antifungal, anti-inflammatory, and anti-atherosclerotic have been extensively studied (Argoudelis and Zieserl, 1966; Komai, 2003; Lee et al., 2011; Zhou et al., 2017; Wu et al., 2019). Here, we report for the first time that asperlin (**5**) exhibits an *in vivo* antifungal activity against TLB and WLR.

## Conclusion

Evaluation of antagonistic microbes is vital for a better understanding of the ecological significance of the biocontrol of plant diseases. In this study, our results showed that *A. candidus* SFC20200425-M11 and *A. montenegroi* SFC20200425-M27 isolated from a marine environment exhibit a biocontrol potential for the first time. Although a variety of secondary metabolites and their biological activities have been reported from *A. candidus*, there is limited information on *A. candidus* and its secondary metabolites in plant disease control efficacy. Given that *A. candidus* and *A. montenegroi* culture filtrates effectively control various plant diseases, we isolated and identified two new compounds (**4** and **6**), along with five known compounds (**1–3**, **5**, and **7**). The *in vitro* results revealed the broad antifungal spectrum of sphaeropsidin A (**1**), (*R*)-formosusin A (**2**), (*R*)-variotin (**3**), and asperlin (**5**), and these natural compounds exhibited plant disease control efficacies. In addition to the antifungal activity, asperlin (**5**) exclusively showed an antibacterial activity against *C. michiganensis* and *E. amylovora*. Taken together, our results suggest that *Aspergillus* spp. and their antimicrobial compounds have great potential to be developed as new biocontrol agents or used as active ingredients for natural pesticides in agricultural fields.

## Data Availability Statement

The original contributions presented in the study are included in the article/Supplementary Material; further inquiries can be directed to the corresponding authors.

## Author Contributions

HK and GC provided the study idea and supervision. MN, BK, YK, and MP contributed to the experimental performance. MN, JH, and HK contributed to the manuscript preparation. MN, JH, HK, and GC contributed to the structure elucidation, revising, and proofreading of the manuscript. All authors contributed to the article and approved the submitted version.

## Funding

This study was supported by the Cooperative Research Program for Agricultural Science and Technology Development (project PJ016028), Rural Development Administration, Republic of Korea.

## Conflict of Interest

The authors declare that the research was conducted in the absence of any commercial or financial relationships that could be construed as a potential conflict of interest.

## Publisher’s Note

All claims expressed in this article are solely those of the authors and do not necessarily represent those of their affiliated organizations, or those of the publisher, the editors and the reviewers. Any product that may be evaluated in this article, or claim that may be made by its manufacturer, is not guaranteed or endorsed by the publisher.

## Footnotes

^1^ http://www.ncbi.nlm.nih.gov
