# Supplementary material for: Biocontrol Potential of Aspergillus Species Producing Antimicrobial Metabolites
Source: Front Microbiol. 2021 Dec 23;12:804333. doi: 10.3389/fmicb.2021.804333 (PMC8733401; doi:10.3389/fmicb.2021.804333)
Supplement: Supplementary file 1 [file Data_Sheet_1.PDF]

Biocontrol Potential of *Aspergillus* Species Producing Antimicrobial Metabolites

Men Thi Ngo[1,2], Minh Van Nguyen[1,2], Jae Woo Han[1], Bomin Kim[1,2], Yun Kyung Kim[1,2], Myung Soo Park[3], Hun Kim[1,2,]* and Gyung Ja Choi[1,2,]*

[1] *Center for Eco-friendly New Materials, Korea Research Institute of Chemical Technology, Daejeon, Korea*

[2] *Department of Medicinal Chemistry and Pharmacology, University Science and Technology, Daejeon, Korea*

[3] *Department of School of Biological Sciences, Seoul National University, Seoul, Korea*

* Correspondence to: Hun Kim (E-mail: hunkim@krict.re.kr) and Gyung Ja Choi (E-mail: kjchoi@krict.re.kr)

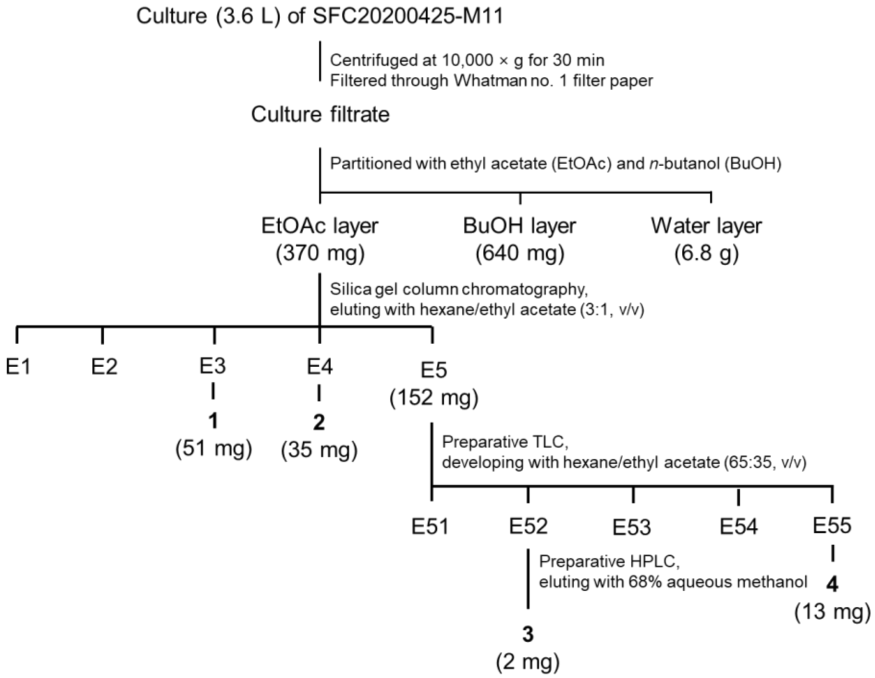

Figure S1. Isolation scheme of compounds **1**–**4** from the culture broth of *Aspergillus candidus* SFC20200425-M11.

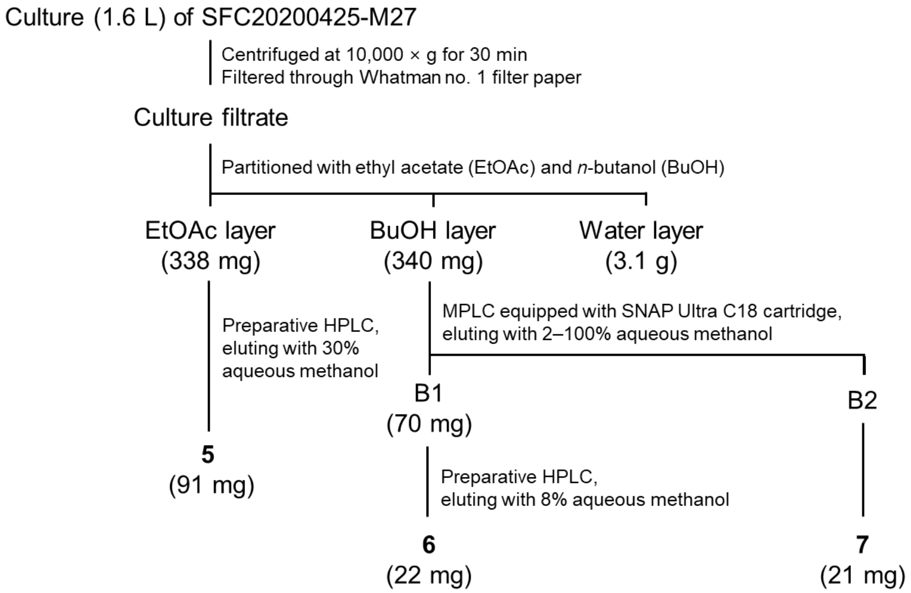

Figure S2. Isolation scheme of compounds **5**–**7** from the culture broth of *Aspergillus montenegroi* SFC20200425-M27.

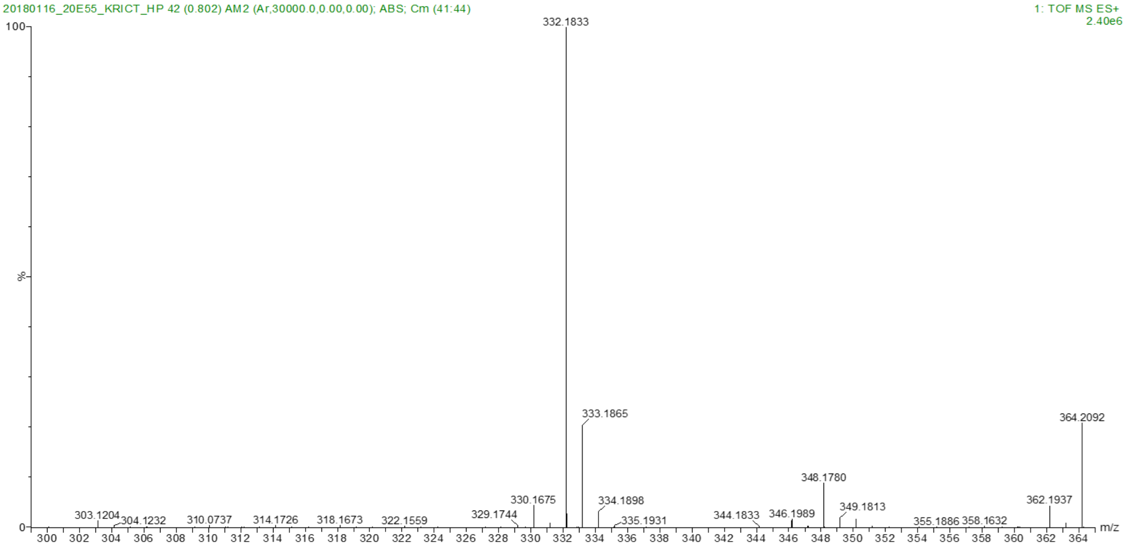

Elemental Composition Report

Single Mass Analysis
Tolerance = 5.0 PPM / DBE: min = -1.5, max = 50.0
Element prediction: Off
Number of isotope peaks used for i-FIT = 3

Monoisotopic Mass, Even Electron Ions
19 formula(e) evaluated with 1 results within limits (all results (up to 1000) for each mass)
Elements Used:
C: 1-20 H: 1-30 N: 1-2 O: 1-5 Na: 1-1

Minimum:				-1.5				
Maximum:		100.0	5.0	50.0				
Mass	Calc. Mass	mDa	PPM	DBE	i-FIT	Norm	Conf(%)	Formula
332.1833	332.1838	-0.5	-1.5	4.5	1073.7	n/a	n/a	C17 H27 N O4 Na

Figure S3. The positive HRESIMS spectrum of compound **4**.

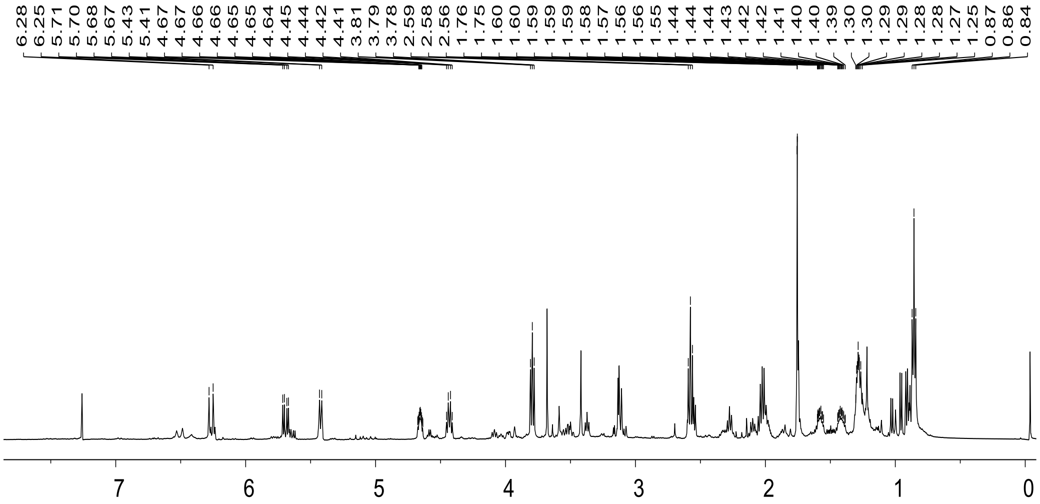

Figure S4. The ^{1}H-NMR spectrum (500 MHz, chloroform-*d*) of compound **4**.

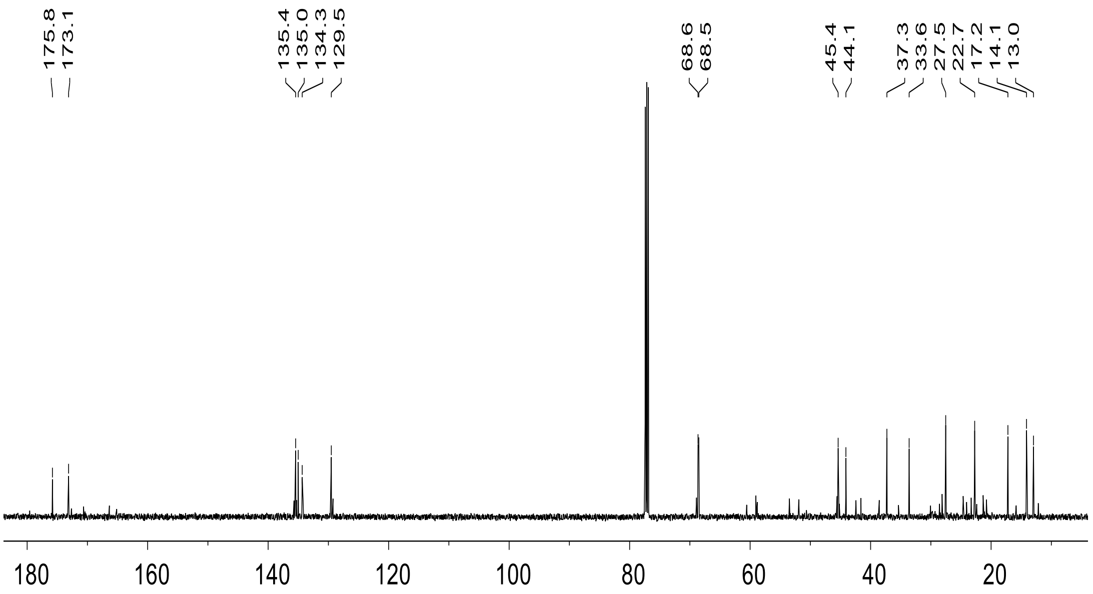

Figure S5. The ^{13}C-NMR spectrum (125 MHz, chloroform-*d*) of compound **4**.

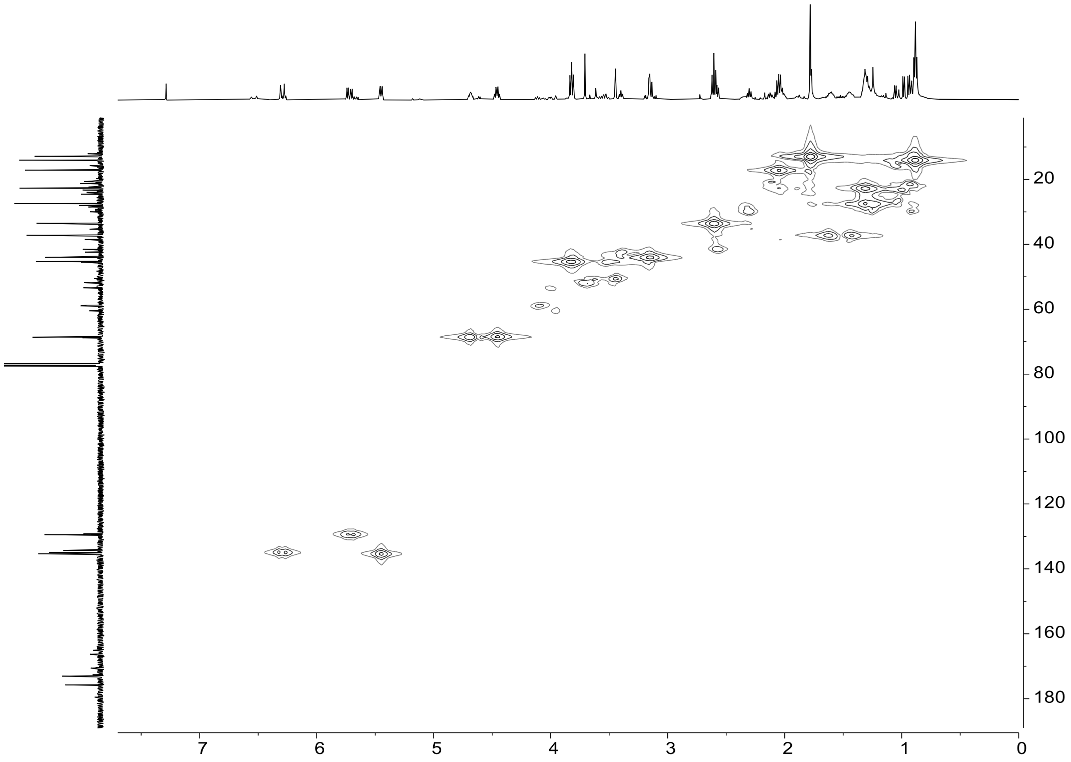

Figure S6. The HSQC spectrum (500 MHz, chloroform-*d*) of compound **4**.

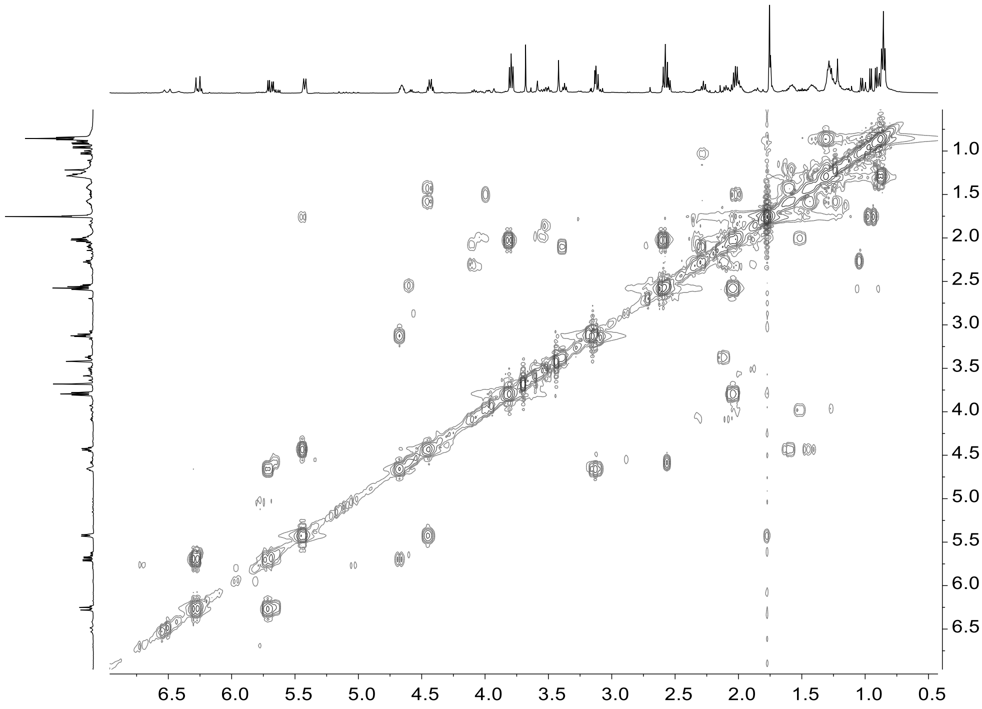

Figure S7. The ^{1}H–^{1}H COSY spectrum (500 MHz, chloroform-*d*) of compound **4**.

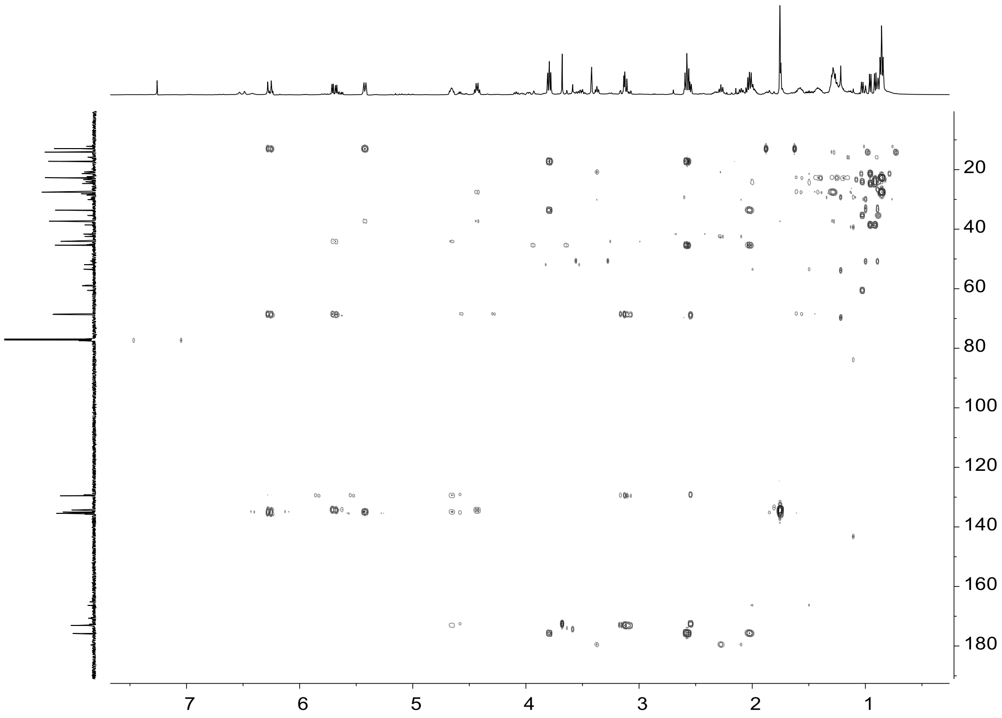

Figure S8. The ^{1}H–^{13}C HMBC spectrum (500/125 MHz, chloroform-*d*) of comopund **4**.

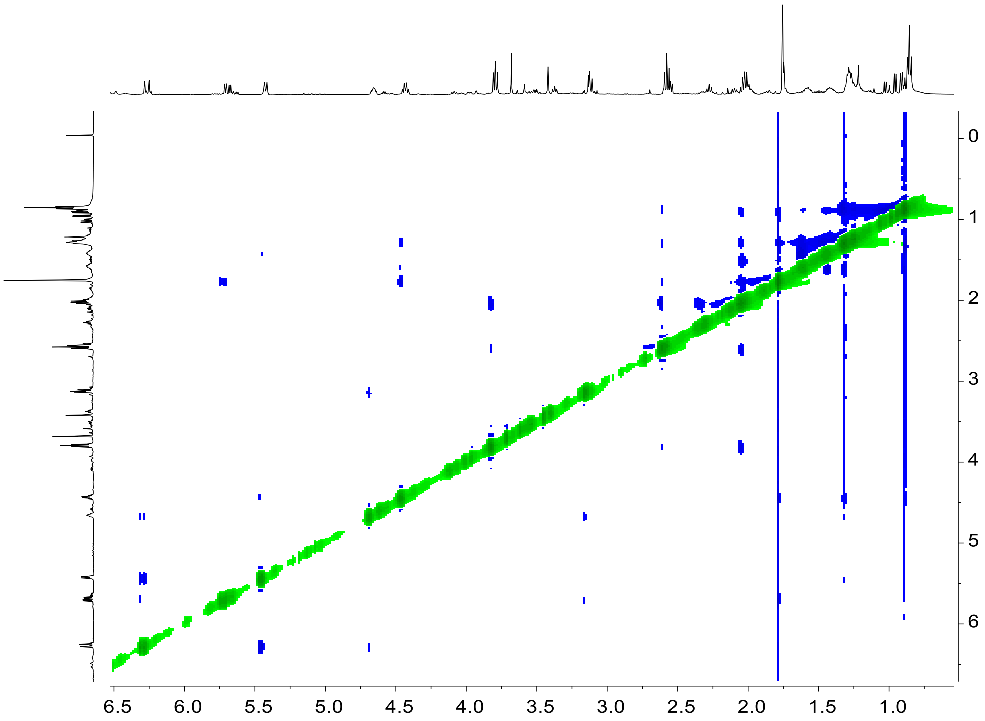

Figure S9. The NOESY spectrum (500 MHz, chloroform-*d*) of compound **4**.

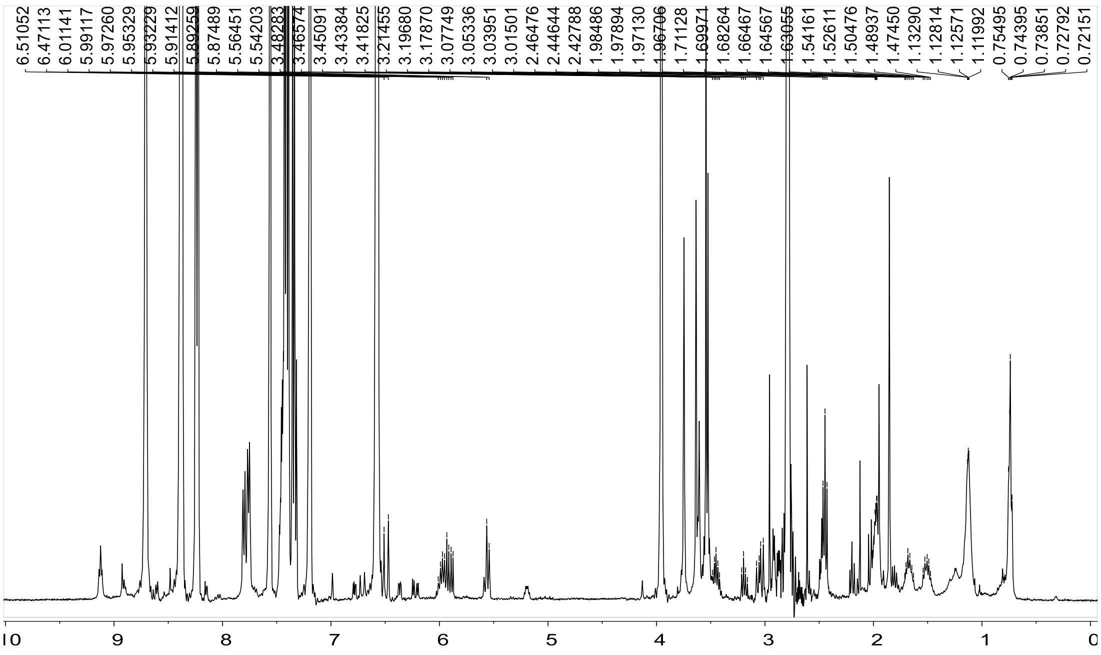

Figure S10. The ^{1}H-NMR spectrum (500 MHz, pyridine-d_5) for the *R*-MTPA ester of compound **4** (**4a**) .

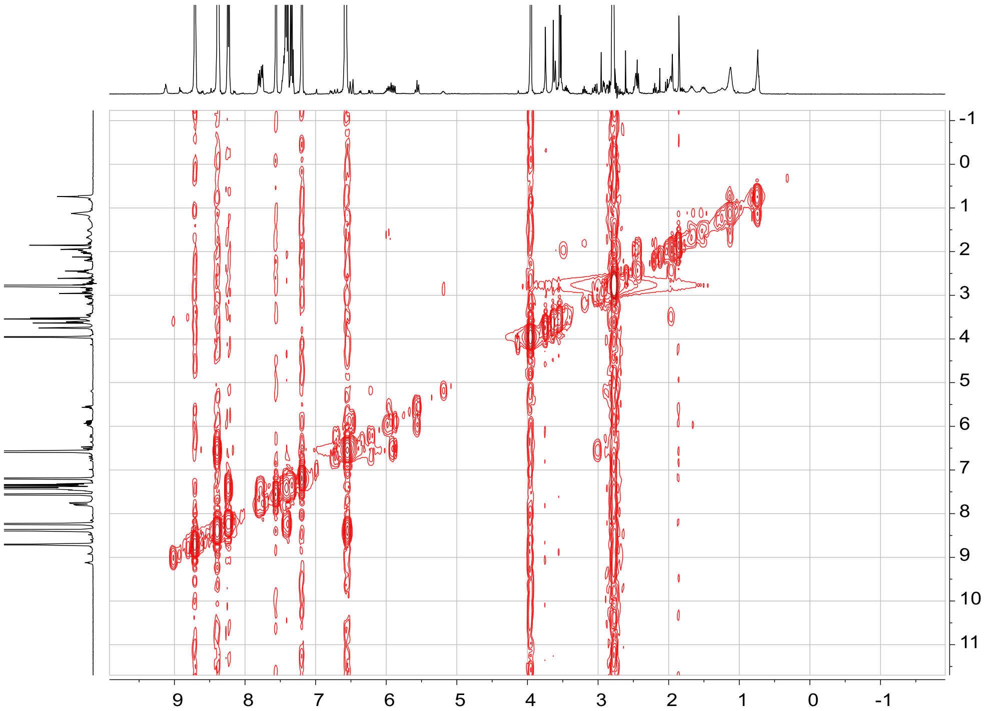

Figure S11. The ^{1}H–^{1}H COSY spectrum (500 MHz, pyridine-d_5) for the *R*-MTPA ester of compound **4** (**4a**).

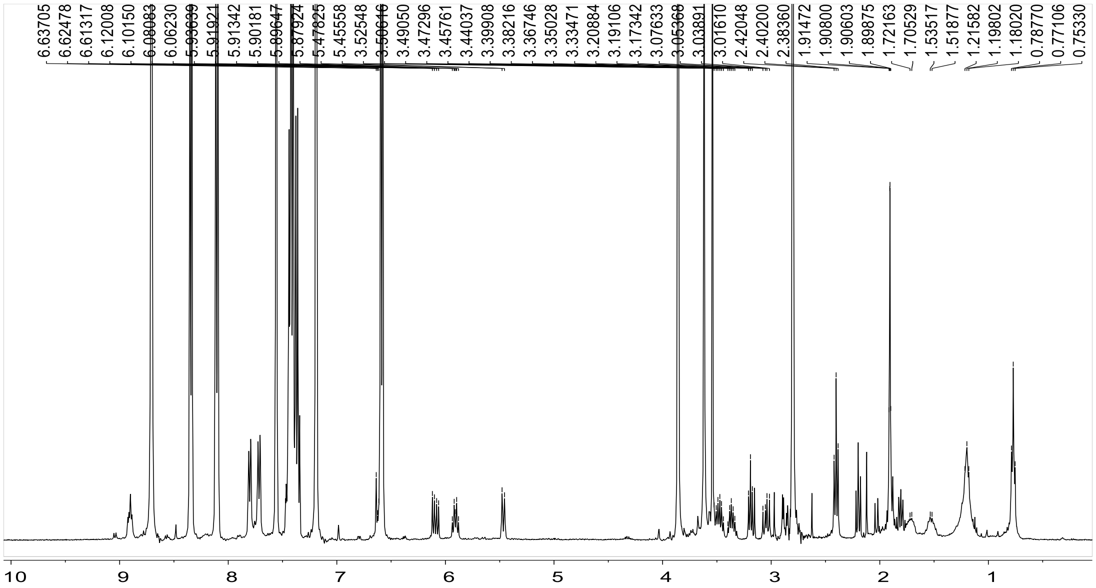

Figure S12. The ^{1}H-NMR spectrum (500 MHz, pyridine-d_5) of *S*-MTPA ester of compound **4** (**4b**).

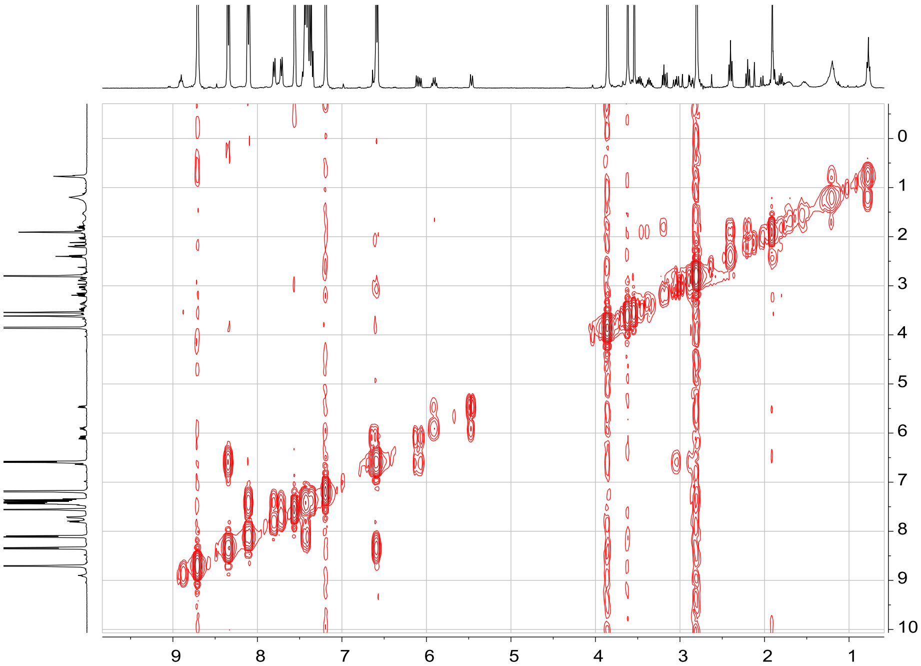

Figure S13. The ^{1}H–^{1}H COSY spectrum (500 MHz, pyridine-d_5) of *S*-MTPA ester of compound **4** (**4b**)

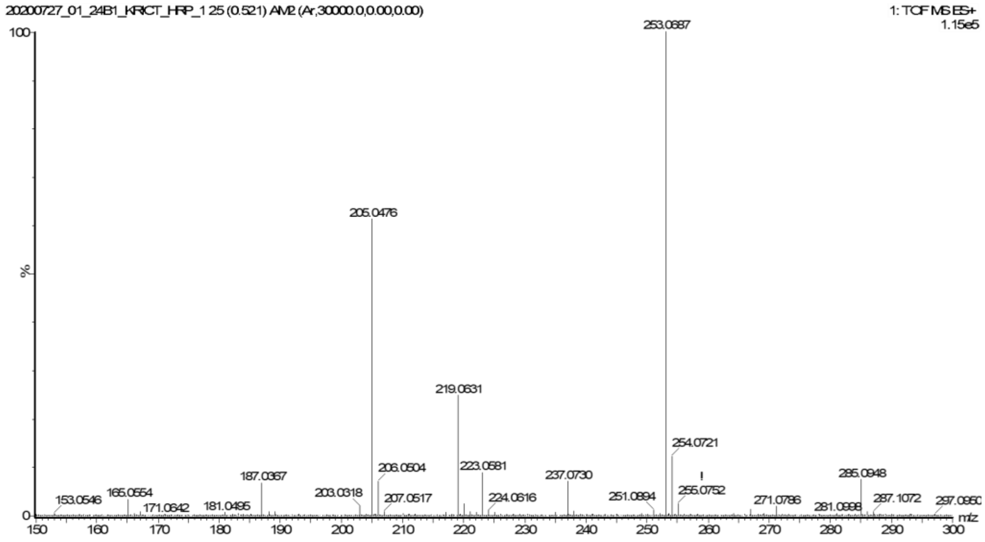

Monoisotopic Mass, Even Electron Ions
Elements Used:
C: 0-50 H: 0-50 O: 0-10 Na: 0-1

Mass	Calc. Mass	mDa	PPM	DBE	i-FIT	Norm	Conf(%)	Formula
Minimum:				-1.5				
Maximum:		100.0	5.0	100.0				
223.0581	223.0582	-0.1	-0.4	3.5	340.5	n/a	n/a	C9 H12 O5 Na
205.0476	205.0477	-0.1	-0.5	4.5	760.9	n/a	n/a	C9 H10 O4 Na

Figure S14. The positive HRESIMS spectrum of compound **6**.

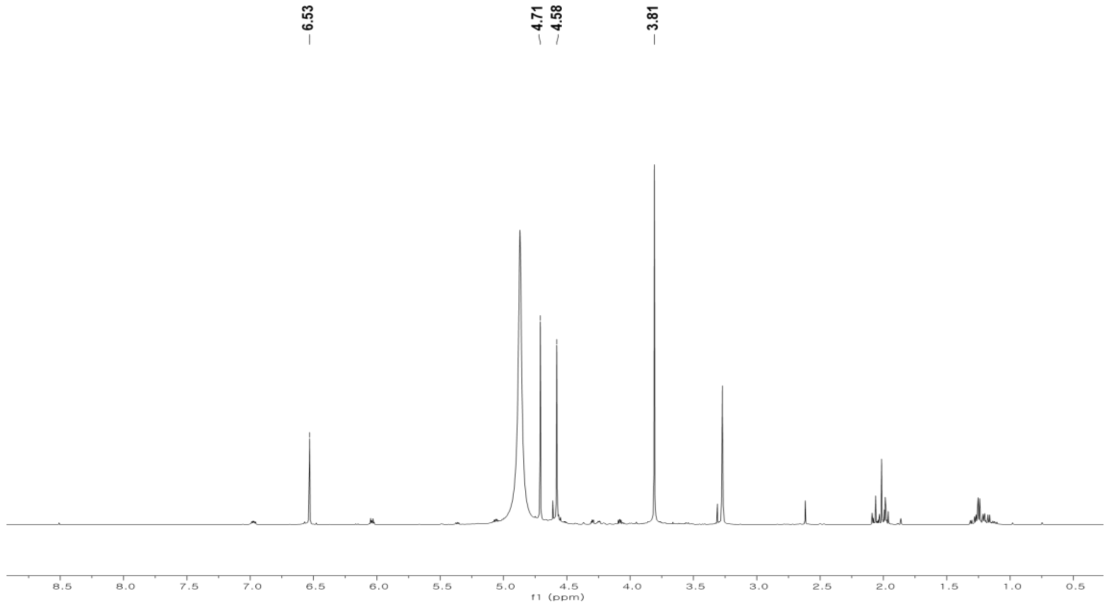

Figure S15. The ^{1}H-NMR spectrum (500 MHz, methanol-d_4) of compound **6**.

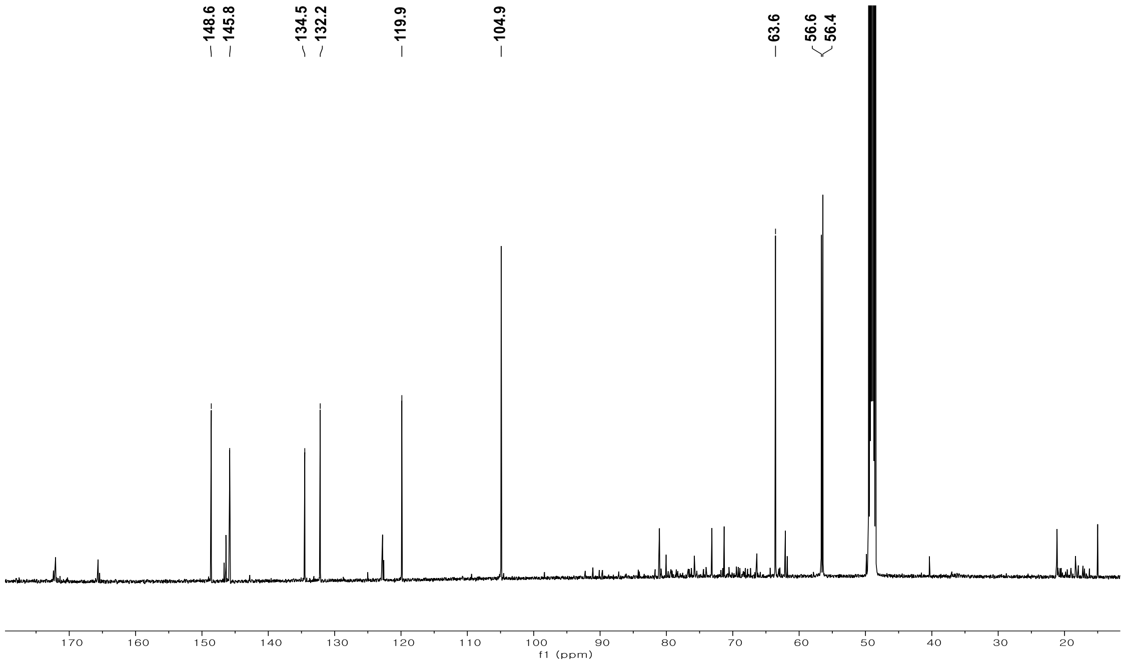

Figure S16. The ^{13}C-NMR spectrum (500 MHz, methanol-d_4) of compound **6**.

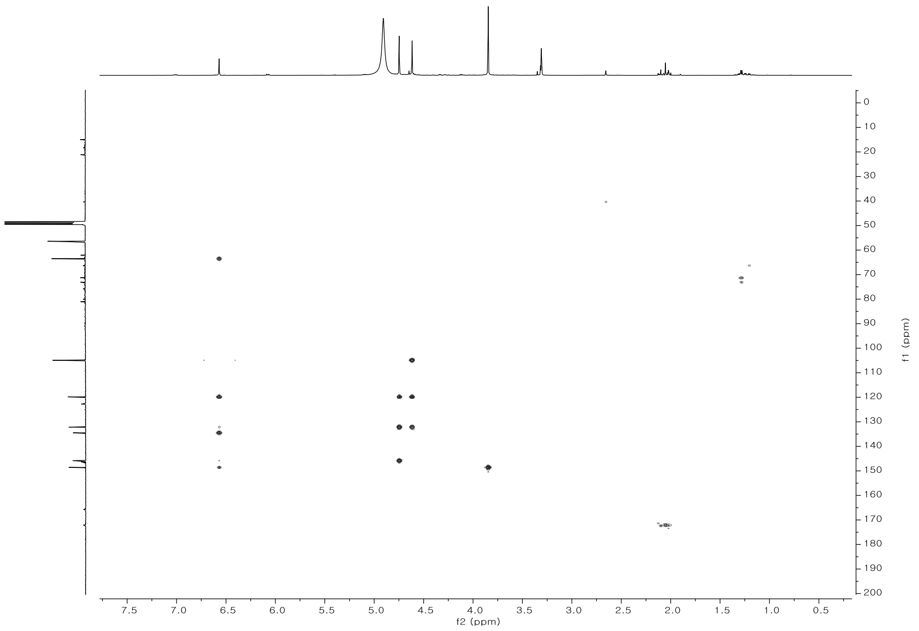

Figure S17. The ^{1}H–^{13}C HMBC spectrum (500 MHz, methanol-d_4) of compound **6**.

Table S1. Summary of *Aspergillus* strains used in this study

species	strain	Site	Source	Reference
A. caesiellus	SFC20160112-M06	Beopsan, Taean, Chungcheongnamdo	Mud flat	Lee et al., 2016
A. candidus	SFC20200425-M11	Jeongam, Yangyang, Gangwondo	Sailfin sandfish egg masses	In this study
A. jensenii	SFC20160610-M03	Chodo, Goseong, Gangwondo	Sailfin sandfish egg masses	In this study
A. luchuensis	SFC20160907-M26	Dongdeok, Gangneung, Gangwondo	Sailfin sandfish egg masses	In this study
A. montenegroi	SFC20200425-M27	Haksan, Suncheon, Jeollanamdo	Mud flat	In this study
A. montevidensis	SFC20160317-M26	Sojuk, Haenam, Jeollanamdo	Mud flat	In this study
A. venenatus	SFC20160407-M10	Oryu, Muan, Jeollanamdo	Sea sand	Lee et al., 2016
A. welwitschiae	SFC20160317-M19	Sojuk, Haenam, Jeollanamdo	Seaweed	In this study

Table S2. The spectroscopic data for known compounds **1**–**3**, **5**, and 7.

**Compound**	**Description**
sphaeropsidin A (**1**)	^{1}H-NMR (500 MHz, chloroform-*d*): δ_H 6.81 (1H, s), 5.88 (1H, dd, 17.5, 10.6 Hz), 5.26 (1H, s), 5.07 (2H, m), 3.44 (1H, s), 2.71 (1H, s), 1.85 (3H, m), 1.60 (4H, m), 1.17 (3H, s), 1.17 (3H, s), and 1.07 (3H, s); ^{13}C-NMR (125 MHz, chloroform-*d*): δ_C 191.8, 174.9, 152.7, 144.4, 133.0, 113.4, 103.8, 71.0, 51.2, 51.3, 40.4, 39.5, 32.6, 32.3, 29.5, 26.9, 24.4, 22.9, 22.5, and 18.1.
(R)-formosusin A (**2**)	^{1}H-NMR (500 MHz, methanol-d_4): δ_H 7.56 (1H, dd, 15.6, 11.5 Hz), 6.99 (1H, d, 11.4 Hz), 6.77 (1H, t, 11.4 Hz), 6.65 (1H, d, 15.4 Hz), 5.69 (1H, d, 8.7 Hz), 4.49 (1H, dt, 8.8, 6.6 Hz), 3.86 (2H, m), 2.63 (2H, t, 8.1 Hz), 2.05 (2H, m), 1.89 (3H, m), 1.63 (1H, m), 1.48 (1H, m), 1.37 (3H, m), 1.30 (1H, m), and 0.94 (3H, t, 7.0 Hz); ^{13}C-NMR (125 MHz, methanol-d_4): δ_C 177.4, 167.6, 148.0, 146.4, 141.3, 135.9, 126.6, 118.8, 69.0, 46.9, 38.2, 34.6, 28.7, 23.7, 18.0, 14.4, and 13.0.
(R)-variotin (**3**)	^{1}H-NMR (500 MHz, chloroform-*d*): δ_H 7.51 (1H, dd, 15.0, 10.9 Hz), 7.38 (1H, d, 15.3 Hz), 6.61 (1H, t, 15.3 Hz), 6.47 (1H, dd, 15.3, 10.9 Hz), 5.66 (1H, d, 8.6 Hz), 4.51 (1H, q, 7.1 Hz), 3.88 (2H, m), 2.62 (2H, dd, 9.2, 7.0 Hz), 2.05 (2H, m), 1.85 (3H, m), 1.66-1.25 (6H, m), and 0.90 (3H, t, 7.0 Hz).
asperlin (**5**)	^{1}H-NMR (500 MHz, methanol-d_4): δ_H 7.15 (1H, dd, 9.7, 5.8 Hz), 6.27 (1H, d, 9.7 Hz), 5.45 (1H, dd, 5.8, 2.8 Hz), 4.48 (1H, dd, 5.9, 2.8 Hz), 3.20 (1H, qd, 5.2, 2.1 Hz), 3.09 (1H, dd, 5.9, 2.1 Hz), 2.17 (3H, s), and 1.40 (3H, d, 5.2 Hz); ^{13}C-NMR (125 MHz, methanol-d_4): δ_C 171.3, 163.8, 142.2, 125.4, 79.3, 63.3, 56.6, 54.6, 20.4, and 17.3.
Protulactone A (**7**)	$[\alpha]_D^{23.5}$ = +6 (*c* 0.34, methanol); ^{1}H-NMR (500 MHz, DMSO-d_6): δ_H 4.93 (1H, dd, 6.6, 4.2 Hz), 4.75 (1H, d, 4.1 Hz), 4.72 (1H, t, 4.6 Hz), 4.16 (1H, d, 4.7 Hz), 3.73 (1H, t, 4.5 Hz), 2.87 (1H, dd, 18.0, 5.0 Hz), 2.45 (1H, d, 18.0 Hz), 1.95 (3H, s), and 1.14 (3H, d, 6.5 Hz); ^{13}C-NMR (125 MHz, DMSO-d_6): δ_C 175.4, 169.8, 90.1, 88.2, 77.6, 75.1, 69.0, 36.0, 20.9, and 15.6.

Table S3. Disease control efficacy of compounds **1**, **2** and **5** isolated from *Aspergillus* species against plant diseases including rice blast (RCB), tomato gray mold (TGM), tomato late blight (TLB), barley powdery mildew (BPM), and pepper anthracnose (PAN).

Treatment	Conc. (μg/mL)	Disease control (%)[a]					
		RCB	TGM	TLB	WLR	BPM	PAN
sphaeropsidin A (**1**)	500	38±18	14±0	96±0	90±5	42±12	10±14
	250	0	14±0	96±0	53±5	8±12	5±7
	125	0	0±0	94±2	13±0	8±12	5±7
(*R*)-formosusin A (**2**)	500	0	82±5	0	60±9	0	10±14
	250	0	57±0	0	3±5	0	0
	125	0	21±10	0	0	0	0
asperlin (**5**)	500	0	0	95±6	95±3	42±12	0
	250	0	0	95±2	83±2	0	0
	125	0	0	90±3	53±1	0	0
